# A20 negatively regulates necroptosis-induced microglia/macrophages polarization and mediates cerebral ischemic tolerance via inhibiting the ubiquitination of RIP3

**DOI:** 10.1038/s41419-024-07293-2

**Published:** 2024-12-18

**Authors:** Meiqian Qiu, Wenhao Zhang, Jiahua Dai, Weiwen Sun, Meijing Lai, Shiyi Tang, En Xu, Yuping Ning, Lixuan Zhan

**Affiliations:** 1 Department of Neurology, Institute of Neuroscience, Key Laboratory of Neurogenetics and Channelopathies of Guangdong Province and the Ministry of Education of China, The Second Affiliated Hospital, Guangzhou Medical University, Guangzhou, China; 2 The Affiliated Brain Hospital of Guangzhou Medical University, Guangzhou, China; 3 Key Laboratory of Neurogenetics and Channelopathies of Guangdong Province and the Ministry of Education of China, Guangzhou Medical University, Guangzhou, China

**Keywords:** Stroke, Cell death in the nervous system, Necroptosis, Acute inflammation

## Abstract

Neuronal necroptosis appears to be suppressed by the deubiquitinating enzyme A20 and is capable to regulate the polarization of microglia/macrophages after cerebral ischemia. We have demonstrated that hypoxic preconditioning (HPC) can alleviate receptor interacting protein 3 (RIP3)-induced necroptosis in CA1 after transient global cerebral ischemia (tGCI). However, it is still unclear whether HPC serves to regulate the phenotypic polarization of microglia/macrophages after cerebral ischemia by mitigating neuronal necroptosis. We hence aim to elucidate the underlying mechanism(s) by which the ubiquitination of RIP3-dependent necroptosis regulated by A20 affects microglia/macrophages phenotype after cerebral ischemic tolerance. We found that microglia/macrophages in CA1 of rats underwent M1 and M2 phenotypic polarization in response to tGCI. Notably, the treatment with HPC, as well as inhibitors of necroptosis, including Nec-1 and mixed lineage kinase domain-like (MLKL) siRNA, attenuated neuroinflammation associated with M1 polarization of microglia/macrophages induced by tGCI. Mechanistically, HPC was revealed to upregulate A20 and in turn enhance the interaction between A20 and RIP3, thereby reducing K63-linked polyubiquitination of RIP3 in CA1 after tGCI. Consequently, RIP3-dependent necroptosis and the M1 polarization of microglia/macrophages were blocked either by HPC or via overexpression of A20 in neurons, which ultimately mitigated cerebral injury in CA1 after tGCI. These data support that A20 serves as a crucial mediator of microglia/macrophages polarization by suppressing neuronal necroptosis in a RIP3 ubiquitination-dependent manner after tGCI. Also, a novel mechanism by which HPC functions in cerebral ischemic tolerance is elucidated.

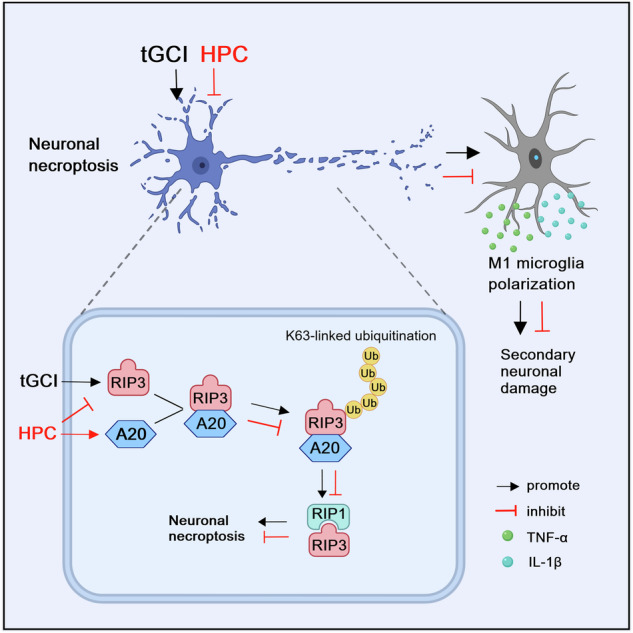

## Introduction

Transient global cerebral ischemia (tGCI) that results from severe hypotension, drowning, or cardiac arrest, often leads to delayed neuronal death in hippocampal cornu amonis (CA) 1 area. Cerebral ischemic preconditioning protects the CA1 pyramidal neurons from severe damage secondary to the ischemic events [1]. Our previous studies have shown that hypoxic preconditioning (HPC) with 8% O_2_ for 30–120 min at 1–4 days before tGCI reduced neuronal death in CA1 of rats [2]. It is, hence, significance to unravel the molecular mechanism of HPC-mediated cerebral ischemia tolerance, which points to novel strategies for preventing and treating cerebral ischemic injuries.

Inflammatory response is deemed to be an integral part of the pathophysiological process in cerebral ischemia, and the inhibition of inflammatory response can reduce neurological deficits after cerebral ischemia [3]. It is well established that the activation of microglia/macrophages, the innate immune effector cells in the central nervous system (CNS), is involved in the robust and persistent neuroinflammatory response after ischemic injury [4]. Under physiological conditions, microglia/macrophages appear to be a “resting phenotype” [5]. After cerebral ischemia, they can be rapidly activated and polarized towards two main phenotypes [6], the former being classically activated M1 microglia/macrophages, which secrete pro-inflammatory cytokines such as tumor necrosis factor-alpha (TNF-α) and interleukin 1 beta (IL-1β), and then mediate the inflammatory cascade reaction, aggravating tissue damage. The latter is alternatively activated M2 microglia/macrophages, which produce anti-inflammatory factors, such as interleukin 10 (IL-10), arginase-1 (Arg-1) and cluster of differentiation 206 (CD206), promoting tissue repair [7–9]. Moreover, the two polar phenotypes often overlap and can be transformed by both intracellular and extracellular factors. Hence, it is an essential target to regulate the polarization of microglia/macrophages for controlling the neuroinflammatory response and alleviating ischemic brain injury. We have reported that HPC can significantly suppress the activation of microglia as well as reduce neuronal damage in CA1 of rats after tGCI [10]. Previous studies have reported that hypoxic pretreatment can promote the polarization of microglia to M2 phenotype and alleviate inflammation induced by middle cerebral artery occlusion [11]. Besides, hypothermia has also been shown to reduce the number of M1 microglia and increase M2 microglia, thereby alleviating ischemic brain injury [12]. This led us to investigate whether the neuroprotective effect of HPC is exerted through modulating the polarization of microglia/macrophages after tGCI.

Necroptosis, a form of inflammatory programmed cell death, can results in the release of inflammatory contents. Recently, the role of necroptosis in neurological diseases, including cerebral ischemia, has drawn much attention [13–15]. The serine/threonine kinase, receptor-interacting protein 1 (RIP1), RIP3, and mixed lineage kinase domain-like (MLKL) constitute the key components in necroptotic signaling pathway [16]. Necroptosis is a strong trigger of innate and adaptive immune responses by releasing damage-associated molecular patterns (DAMPs) [17]. Moreover, DAMPs released from necroptotic tumor cells results in M1 macrophage polarization [18]. Notably, necroptotic neurons can regulate the polarization of microglia/macrophages after cerebral ischemia. For instance, defective necroptosis mediated by knockdown of RIP3 or MLKL contributed to the polarization of microglia/macrophages toward M2 phenotype in the ischemic cortex [19]. And even more strikingly, our previous studies have demonstrated the involvement of necroptosis in neuronal death in CA1 after tGCI, which can be alleviated by HPC [14]. Hence, it is logical to ascertain whether the mitigation of neuronal necroptosis can regulate the phenotypic polarization of microglia/macrophages during HPC-induced cerebral ischemia tolerance.

Necroptotic signaling pathway are known to be tightly regulated by post-translational modifications, especially through ubiquitylation [20]. Particularly, dysregulation of ubiquitylation in necroptotic signaling pathway is closely associated with various inflammatory, infectious [21] and degenerative diseases [22]. Recent evidence also richly supports that RIP3 ubiquitination is involved in necroptosis propagation [23–25]. For example, deubiquitinating enzyme A20 was shown to inhibit the K63-linked polyubiquitination lysine residues of RIP3 and diminish the interaction of RIP1 and RIP3, thereby suppressing necroptosis [25]. A20 (tumor necrosis factor ɑ-induced protein 3, TNFAIP3) is a ubiquitin-editing enzyme that has both ubiquitin ligase and deubiquitinase activities [26, 27]. Abnormal expression and/or function of A20 has been associated with chronic inflammation and tissue damage, contributing to the immunopathology of multiple human autoimmune and inflammatory diseases [28–30]. Moreover, A20 was shown to contribute to the regulation of microglia/macrophage polarization [31, 32], with recent evidence suggesting the critical role of A20 in regulating necroptosis [23, 33, 34]. Therefore, we hypothesize that HPC upregulates A20, thereby suppressing necroptosis by inhibiting the K63-linked polyubiquitination lysine residues of RIP3, which leads to the polarization of microglia/macrophages after tGCI.

In this study, we aim to explore the role of necroptosis in microglia/macrophages polarization and its contribution to neuroprotection mediated by HPC against tGCI. We also intend to elucidate the underlying mechanism(s) by which RIP3 ubiquitination-dependent necroptosis regulated by A20 affects microglia/macrophages phenotype, in order to further our understanding of pathogenesis of ischemic stroke and potentially provide new therapeutic targets for cerebral ischemia.

## Materials and methods

### Animals

Experiments were performed on adult male Wistar rats weighing 220–280 g (aged 7–8 weeks; Southern Medical University, Guangzhou, China). Rats were housed in a temperature-controlled (22–26 °C) and 12-h light/dark cycle environment with ad libitum access to food and water. All surgical operation and animal experiments were conducted according to the guidelines of Animal Research: Reporting In Vivo Experiments (ARRIVE) under the oversight of the Animals Care and Use Committee of Guangzhou Medical University (Guangzhou, China). Every effort had been made to minimize the number of animals used and to reduce animal suffering and distress. Animals went through randomization using a random number table and were divided into different groups according to the standard procedures.

Based on our and others’ published studies and the results from our preliminary experiments in this study, the required sample size was estimated. In total, 437 rats were used. Nine rats in tGCI groups and five in HPC groups succumbed to the tGCI procedure. Four rats in tGCI groups and three in HPC groups died after tGCI. Additionally, Thirteen rats died after intracranial injection. Four died during anesthesia, and three died during the hypoxia procedure. Three rats that presented with convulsion after ischemia were also excluded.

### Transient global cerebral ischemia and hypoxic preconditioning

A four-vessel occlusion method was used to induce tGCI [35]. Animals were anesthetized by inhalation of isoflurane (induction, 3–4%; maintenance, 2–3%). Bilateral vertebral arteries were electrocauterized. Bilateral common carotid arteries were isolated and then a teflon/silastic occluding device was assembled loosely around each common carotid artery without blood flow interruption. After 24 h of surgery, global cerebral ischemia was induced by occluding bilateral common carotid arteries for 10 min in awake rats. The rats with mydriasis and loss of the righting reflex within 1 min were selected for the following experiments, while those convulsed during ischemia or post-ischemia were excluded from this study. Rectal temperature was maintained at 37–38 °C throughout the procedure. All operations were conducted by skilled technicians under aseptic conditions. Rats in sham-operated (Sham) group received the same surgical operations, except the occlusion of the common carotid arteries. In HPC groups, rats were placed in a hypoxic chamber at 24 h before tGCI, through which air containing 8% O_2_ and 92% N_2_ flowing continuously at a temperature of 23–25 °C for 30 min. Twenty-four hours after sham-operated procedures without ischemia, sham-operated, hypoxia-treated rats (hereinafter referred to as hypoxia group) were exposed to 30-min hypoxia.

### Immunohistochemistry

The avidin-biotin-peroxidase complex (ABC) method was used to perform single-label immunohistochemistry. Rats were sacrificed at 0, 4, 24, 48, and 168 h of reperfusion with or without HPC, and were perfused intracardially with normal saline and 4% paraformaldehyde in phosphate-buffered saline (PBS), respectively. The removed whole brains were pre-fixed in 4% paraformaldehyde for 24 h, and then post-fixed in 10%, 20%, and 30% sucrose solutions, successively. Brains were sliced into 30-µm coronal sections using a cryotome (Leica, Wetzlar, Hessen, Germany). The dorsal hippocampus sections (between anterior-posterior (AP) 4.8 and 5.8 mm, interaural or AP 3.3–3.4 mm, bregma) were treated with 3% hydrogen peroxide for 30 min, followed by 5% normal serum for 1 h at room temperature, and then incubated overnight at 4 °C with primary antibodies. The antibodies included mouse anti-A20 (1:100; Abcam, Cambridge, USA) and mouse anti-neuronal nuclei (NeuN; 1:5000; MilliporeSigma, Boston, MA, USA). After being washed with PBS, the sections were incubated with biotinylated secondary immunoglobulin G antibody for 2 h, and treated with ABC for 30 min at room temperature. The peroxidase reaction was visualized with 0.05% diaminobenzidine and 0.01% hydrogen peroxide. The number of immunopositive cells was counted by the total number of four non-repeated random fields (0.037 mm^2^/field × 4 = 0.148 mm^2^ in total) in CA1 under a light microscope with ×660 magnification. In addition, four sections from each rat were evaluated blindedly. A negative control without primary antibody was performed for all experiments.

Double-fluorescent immunohistochemistry was carried out, as described previously, to observe the expression patterns of inducible nitric oxide synthase (iNOS), CD206, RIP3 and A20 in CA1 of different groups. NeuN, glial fibrillary acidic protein (GFAP), and ionized calcium binding adaptor molecule-1 (Iba-1) were used to identify neuronal nuclei, astrocytes, and microglia, respectively. Primary antibodies used in this study included rabbit anti-iNOS (1:200; GeneTex, Irvine, CA, US), goat anti-CD206 (1:200; Novus, Colorado, USA), rabbit anti-RIP3 (1:200; Novus), mouse anti-A20 (1:100; Abcam), mouse anti-Iba-1 (1:200, MilliporeSigma), mouse anti-NeuN (1:1000; MilliporeSigma), mouse anti-GFAP (1:4000; MilliporeSigma), rabbit anti-Iba-1 (1:500; Wako, Osaka, Japan), rabbit anti-NeuN (1:1000; MilliporeSigma), and rabbit anti-GFAP (1:4000; MilliporeSigma). Then, sections were incubated with secondary antibodies at room temperature for 2 h and washed with PBS. The secondary antibodies used in this study included Cy3-conjugated goat anti-mouse IgG H&L (1:100; MilliporeSigma), goat anti-rabbit IgG H&L (Alexa Fluor® 488) (1:100; Abcam), and donkey anti-goat IgG H&L (Alexa Fluor® 488) (1:500; Thermo Fisher Scientific, Waltham, Massachusetts, USA). Finally, slides were observed using a confocal laser microscope (SP8, Leica Microsystems, Wetzlar, Hessen, Germany). A negative control without primary antibody was also included.

### Isolation of total RNA and reverse transcription quantitative real-time polymerase chain reaction (RT-qPCR)

Total RNA was isolated from hippocampal CA1 of brain using Trizol Reagent (Invitrogen, USA) according to the manufacturer’s protocol. Then, extracted RNA was reverse-transcribed into cDNA by a PrimeScript RT reagent Kit (Takara, Tokyo, Japan). RT-qPCR was carried out using the SYBR PremixEx TaqII kit (Takara). The primers used are as follows: TNF-α-F 5′-AAAGGACACCATGAGCACGGAAAG-3′, TNF-α-R 5′-CGCCACGAGCAGGAATGAGAAG-3′, IL-1β-F 5′-GTCTGACCCATGTGAGCTGAA-3′, IL-1β-R 5′-CAAGGCCACAGGGATTTTGTC-3′, IL-10-F 5′-CTGCTCTTACTGGCTGGAGTGAAG-3′, IL-10-R 5′-TGGGTCTGGCTGACTGGGAAG-3′, Arg-1-F 5′-CGGCAGTGGCGTTGACCTTG-3′, Arg-1-R 5′-GTTCTGTTCGGTTTGCTGTGATGC-3′, glyceraldehyde 3-phosphate dehydrogenase (GAPDH)-F 5′-ACGGCAAGTTCAACGGCACAG-3′, and GAPDH-R 5′-CGACATACTCAGCACCAGCATCAC-3′. The procedure of PCR was as follows: pre-denaturation at 95 °C for 30 s, 40 cycles of 95 °C for 3 s, 60 °C for 30 s, and 50 °C for 30 s, and an additional extension at 50 °C for 30 s. All samples were run in triplicate. The data were analyzed using the 2^–ΔΔCt^ method. GAPDH was regarded as an internal reference for relative quantification. The relative expression levels of the mRNAs were then reported as fold changes versus Sham group.

### Western blot

Rats were sacrificed at 0, 4, 24, and 48 h after reperfusion with or without HPC, respectively. The brain tissue was incised into successive 2-mm coronal slices using a brain matrix, and CA1 regions of bilateral hippocampi were quickly isolated under the stereomicroscope. Total proteins of CA1 subregion were extracted as previously described [2]. Bicinchoninic acid (BCA) method was performed to measure protein concentration according to the manufacturer (Beyotime, Jiangsu, China). Western blot was conducted as described previously [2]. In brief, the proteins were separated by 10% or 12% sodium dodecyl sulfate-polyacrylamide gel electrophoresis and then transferred onto polyvinylidene fluoride (PVDF) membranes (MilliporeSigma). The PVDF membranes were blocked in 5% nonfat dried milk at room temperature for 1 h and subsequently probed with specific primary antibodies at 4 °C overnight. The primary antibodies used in this study are as follows: rabbit anti-TNF-α (1:2000; Abcam), rabbit anti-IL-1β (1:3000; GeneTex, Alton, CA, USA), rabbit anti-IL-1β (1:3000; Novus), rabbit anti-IL-10 (1:2000; Abcam), rabbit anti-Arg-1 (1:1000; Cell Signaling Technology, Danvers, Massachusetts, USA), mouse anti-A20 (1:1000, Abcam), rabbit anti-A20 (1:2000; Proteintech); rabbit anti-RIP3 (1:1000, Novus), mouse anti-RIP1 (1:1000, BD, Franklin, New Jersey, USA), rabbit anti-RIP1 (1:2000; Abcam), and mouse anti-GAPDH (1:10000; Proteintech). The quantitative densitometric analysis of the protein bands was carried out with ImageJ (NIH, Bethesda, Maryland, USA). Relative optical densities of protein bands were calibrated with that of GAPDH and normalized to those in Sham rats. A negative control without primary antibody was performed for all experiments.

### Drug or MLKL-siRNA administration

To inhibit necroptosis, necrostatin-1 (Nec-1, 5-(1H-indol-3-ylmethyl)- (2-thio-3-methyl) hydantoin, 5.2 μM, 10 µl; MilliporeSigma) or vehicle (20% Dimethyl sulfoxide (DMSO) in PBS) was administrated as previously described [16]. In short, drugs were intracerebroventricularly administrated at 24 h before tGCI via the cannula affixed to the right parietal skull (1.0 mm posterior to bregma, 1.5 mm lateral to bregma, and 3.6 mm below the dura). To clarify the effect of MLKL on polarization of microglia, MLKL-siRNA or Con-siRNA from RiboBio (Guangzhou, China) was delivered into CA1 regions of bilateral hippocampi (3.5 mm posterior to bregma, 2.3 mm lateral to bregma, and 2.5 mm below the dura) of rats at 24 h before ischemia, as previously described [15]. The sequence of MLKL-siRNA is 5′-GCTACTGTGGGCAGTGATA-3′.

### Immunoprecipitation

Immunoprecipitation was performed as previously described [15]. An amount of 300 µg of extracted protein was incubated with primary antibody against RIP3 (diluted 1:50; Novus) at 4 °C overnight. The next day, the protein-antibody immune complexes were added into packed protein G agarose beads (MilliporeSigma) for 4 h of incubation at 4 °C. Then, the complexes were washed and collected by centrifugation and eluted by boiling in loading buffer. The eluted protein samples were subjected to Western blot with antibodies. The primary antibodies are as follows: rabbit anti-K63-Ub (1:2000; Cell Signaling Technology), mouse anti-A20 (1:1000; Abcam), rabbit anti-RIP3 (1:1000; Novus), and mouse anti-RIP1 (1:1000; BD). Densitometric analysis of the relative precipitated proteins bands was calibrated with the bands of RIP3 (ratio of bound to RIP3) and normalized to those in Sham rats.

### Adeno-associated virus (AAV) construction and administration

Three small-interfering RNA sequences targeting of neurons *A20* (A20, GenBank accession number XM_003748656) and a negative control vector (CON323) were designed by Genechem (Shanghai, China). Briefly, RNA interference (RNAi) was inserted into EcoRI and BamHI sites of the hSyn promoter-EGFP-MIR155 (MCS)-SV40 PolyA (GV680) AAV vector according to the manufacturer’s instruction. The shuttle vector and viral packaging system were cotransfected into AAV-293 cells, which derived from HEK293 cells, to produce recombinant AAV particles. The best-performing A20-RNAi sequence is ACCGCTAGCTAACTGGAGGCTTGCTGAAGGCTGTATGCTGTATTCGCTGGCTTAGGTGCTGGTTTTGG (A20-RNAi) with maximal inhibitory efficacy. Therefore, A20-RNAi was utilized for subsequent experiments. Titers of AAV were 2.25 × 10^13^ TU/ml. A 4-μl volume containing 2.25 × 10^10^ TU of particles was bilaterally injected into CA1 region. Rats were allowed to recover for up to 28 days after AAV injection to ensure sufficient gene expression before being used for subsequent experiments.

Plasmids containing the sequence of rat *A20* targeting of neurons (GenBank accession number XM_003748656), and a negative control vector (CON323) were designed by Genechem (Shanghai, China). Briefly, A20 sequence was inserted into EcoRI and BamHI sites of the hSyn promoter-MCS-EGFP-3FLAG-SV40 PolyA (GV466) AAV vector. Titers of AAV were 5.11 × 10^12^ TU/ml. A 4-μl volume containing 1.533 × 10^10^ TU of particles was injected into CA1 regions of bilateral hippocampi. Rats were allowed to recover for up to 14 days after AAV injection to ensure sufficient gene expression before being used for subsequent experiments.

### Assessment of cellular damage

As done previously, Nissl and NeuN staining were performed to verify the hippocampal cell damage after 168 h of reperfusion. Nissl staining with cresyl violet is a classical staining for the cells in brain tissue, and is performed to observe the morphological structures of the neuronal cells. NeuN (a selective neuronal marker) staining is an immunohistochemical method which indicates neuronal cell bodies, and can be used for neuron quantification. Sections from Nissl and NeuN staining were examined under a light microscope (×660), and cell counts were conducted [36]. Cells in the CA1 pyramidal layer were quantitatively analyzed within three non-repeated rectangular areas of 0.037 mm^2^ in the typical dorsal hippocampus (between anterior-posterior (AP) 4.8 and 5.8 mm, interaural or AP 3.3–3.4 mm, bregma). Data were quantified bilaterally in sections from each brain and assessed blindedly, and four sections per animal were evaluated.

### Statistical analysis

Statistical analysis was performed with the Statistical Package for Social Sciences Software for Windows, version 25.0 (SPSS, Inc., Chicago, IL, USA). All variables were showed as mean ± standard deviation (SD). All data were checked by normal distribution and homogeneity of variance, respectively. When the data were normally distributed, one-way ANOVA or two-way analysis followed by a Least Significant Difference (LSD) or Tamhane’s T2 post-hoc test was applied. When the data were abnormally distributed and the variances were unequal, nonparametric tests were used. All *p* values of less than 0.05 were considered statistically significant.

## Results

### HPC suppresses M1 polarization of microglia/macrophages in CA1 after tGCI

**Fig. 1:**
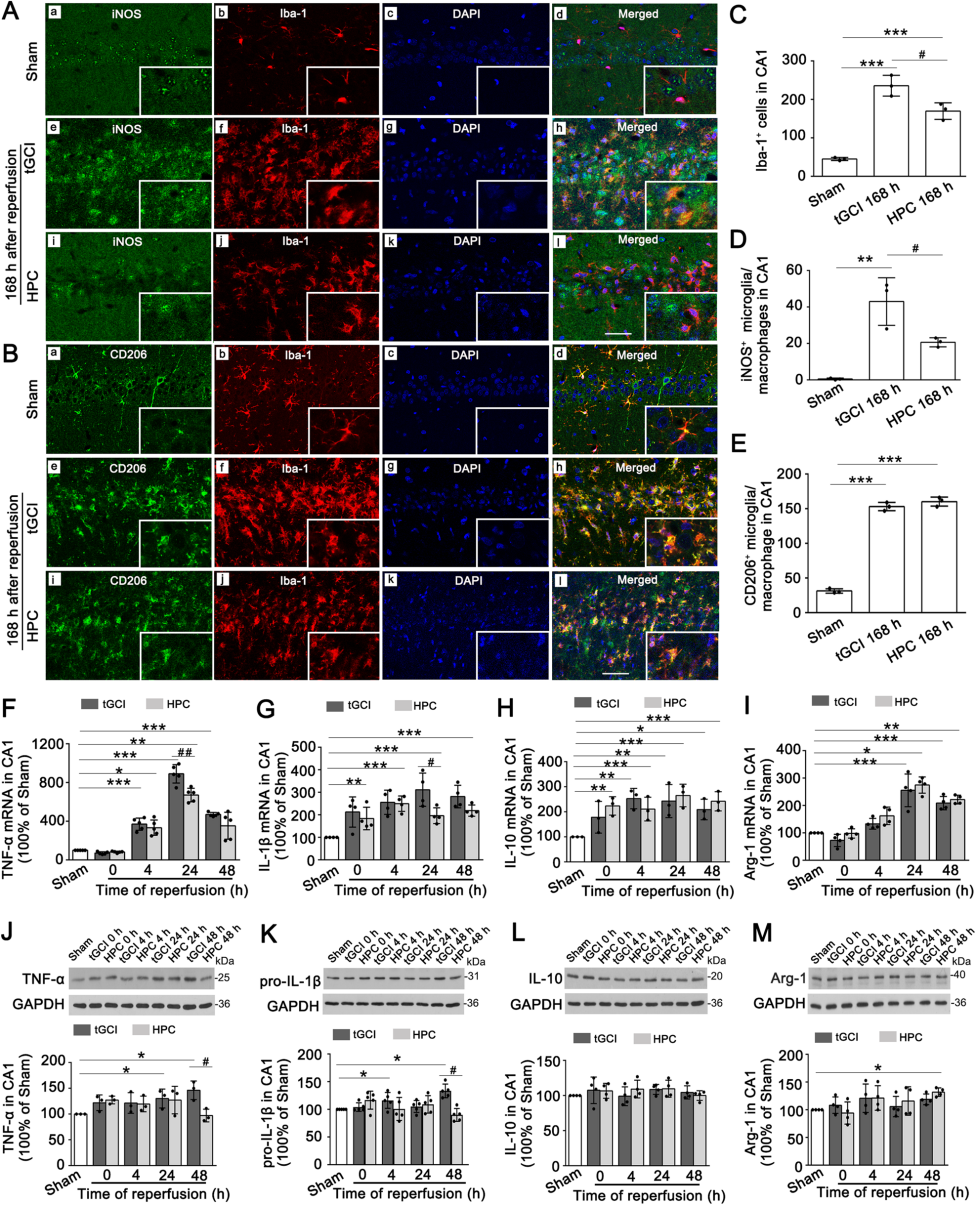
HPC inhibits M1 polarization of microglia/macrophages in CA1 after tGCI. **A**, **B** Representative photomicrographs with fluorescent staining of iNOS/CD206 (green), Iba-1 (red), and DAPI (blue) in CA1 after tGCI with or without hypoxia. Scale bars: 75 μm. **C**–**E** Quantitative analyses of Iba-1^+^ cells, iNOS^+^ and CD206^+^ microglia/macrophages in CA1. HPC decreases the number of Iba-1-positive cells and iNOS^+^ microglia/macrophages in CA1 at 168 h after tGCI. **F**–**I** The histogram presents the qRT-PCR quantitative analyses of the mRNA levels of TNF-α, IL-1β, IL-10 and Arg-1 in CA1. HPC significantly downregulates the mRNA levels of TNF-α and IL-1β in CA1 at 24 h after tGCI. **J**–**M** Representative immunoblots show the expression of TNF-α, pro-IL-1β, IL-10 and Arg-1 in CA1, respectively. The histogram presents the quantitative analyses of TNF-α, pro-IL-1β, IL-10 and Arg-1 in CA1. HPC inhibits the expression of TNF-α and pro-IL-1β in CA1 at 48 h after tGCI. Each bar represents the mean ± S.D. Statistical analysis was performed using ANOVA with LSD or Tamhane’s T2 post-hoc test or Kruskal–Wallis H test. *p < 0.05, **p < 0.01, and ***p < 0.001. NS, no significance.

We firstly detected the activated status and polarization of microglia in CA1 by immunofluorescence assay (Fig. 1A–E). Compared with Sham rats, Iba-1-positive cells became hypertrophic and amoeboid after tGCI with or without hypoxia. Additionally, the co-localization of Iba-1 either with iNOS or CD206 was increased sharply after tGCI with or without hypoxia (Fig. 1A, B). The quantitative analysis showed that the number of Iba-1-positive cells and iNOS-positive microglia/macrophages largely increased at 168 h after tGCI, which could be partly offset by HPC (Fig. 1C, D). An increase of CD206-positive microglia/macrophages was also observed at 168 h after tGCI, whereas, no statistical difference was observed between the tGCI and HPC groups (Fig. 1E).

At the mRNA levels, TNF-α in CA1 were obviously upregulated after 4 h of reperfusion and maintained at this high level until 48 h after tGCI. Meanwhile, the levels of these M1 markers were significantly downregulated in HPC rats at 24 h after reperfusion, compared with tGCI group (Fig. 1F, G). In contrast, M2 markers including IL-10 and Arg-1 also increased after tGCI and maintained at high levels up to 48 h. Nevertheless, HPC didn’t affect the mRNA levels of IL-10 or Arg-1 after tGCI (Fig. 1H, I). The application of Western blot further confirmed the effect of HPC in inhibiting the expression of M1 markers (TNF-α and precursor IL-1β (pro-IL-1β)) at 48 h after tGCI (Fig. 1J, K), rather than on those of M2 (IL-10 and Arg-1) (Fig. 1L, M). However, compared with Sham group, statistical analysis did not reveal a significant effect of hypoxia on the expression of these inflammatory cytokines in CA1 of Sham (Supplementary Fig. S1A–D).

### HPC suppresses M1 polarization of microglia/macrophages via restricting necroptosis in CA1 after tGCI

**Fig. 2:**
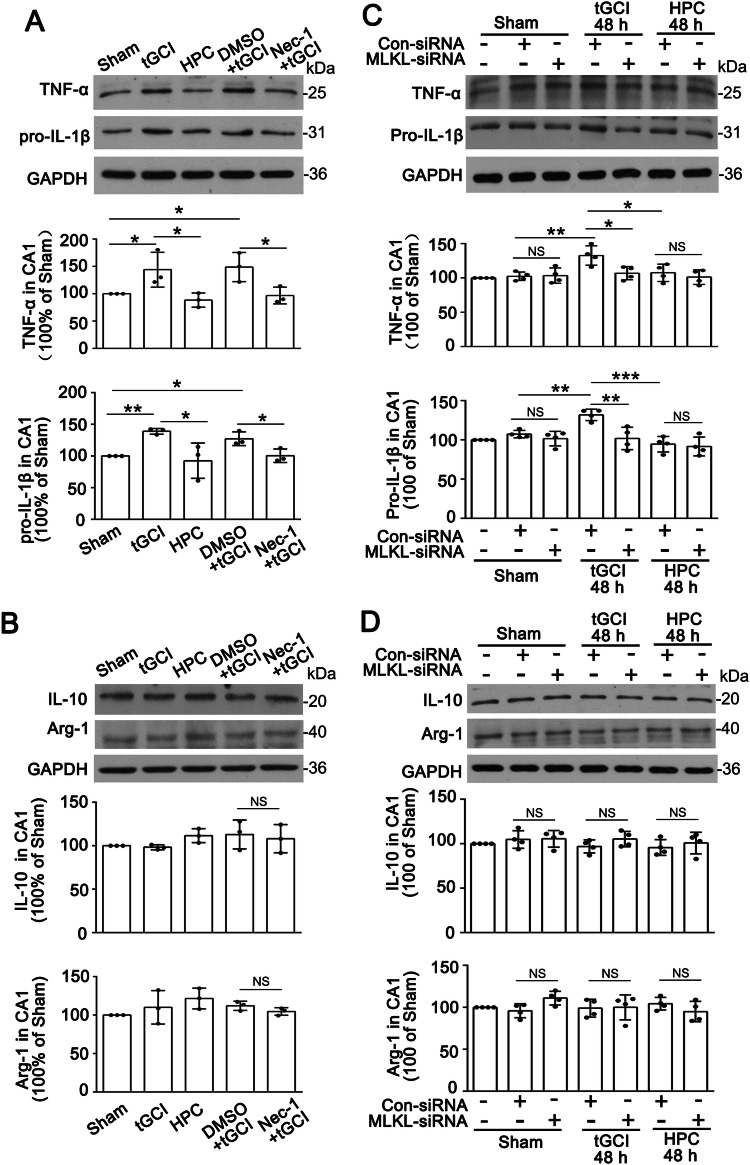
Inhibition of necroptosis by Nec-1 or MLKL-siRNA suppresses M1 polarization of microglia/macrophages in CA1 after tGCI. **A**, **B** Representative images of Western blot show the expression of TNF-α, pro-IL-1β, IL-10, and Arg-1 in CA1 after tGCI with or without Nec-1 treatment. The histogram presents the quantitative analyses of TNF-α, pro-IL-1β, IL-10, and Arg-1 in CA1. Nec-1 diminishes the levels of TNF-α and pro-IL-1β in CA1 after tGCI. Each bar represents the mean ± S.D. Statistical analysis was performed using ANOVA with LSD or Tamhane’s T2 post-hoc test, or Kruskal–Wallis H test. Unpaired *t* test or Mann–Whitney *t* test was used for comparing with tGCI group administrated with DMSO. *p < 0.05, **p < 0.01, and ***p < 0.001. **C**, **D** Representative images of Western blot show the expression of TNF-α, precursor IL-1β, IL-10, and Arg-1 in CA1 after treatment with or with MLKL-siRNA. The histogram presents the quantitative analyses of TNF-α, pro-IL-1β, IL-10, and Arg-1 in CA1. MLKL-siRNA treatment downregulates the expression of TNF-α and pro-IL-1β in CA1 after tGCI. Each bar represents the mean ± S.D. Statistical analysis was performed using ANOVA with LSD or Tamhane’s T2 post-hoc test, or Kruskal–Wallis H test. Unpaired *t* test or Mann–Whitney *t* test was used for comparing with Con-siRNA group. *p < 0.05, **p < 0.01, and ***p < 0.001. NS, no significance.

We further investigated whether necroptosis was involved in M1 polarization of microglia/macrophages in CA1 after tGCI. Firstly, Nec-1, an inhibitor of RIP1 kinase, was injected intracerebroventricularly 24 h before tGCI, to inhibit RIP-mediated necroptosis. The increased levels of TNF-α and pro-IL-1β in CA1 induced by tGCI diminished after Nec-1 treatment (Fig. 2A), while the expression of IL-10 and Arg-1 remained unchanged (Fig. 2B).

MLKL, a crucial downstream molecule of RIP1 and RIP3, has been confirmed as the ultimate executor of necroptosis. Given that HPC can alleviate MLKL-mediated necroptosis [17], we utilized siRNA-mediated knockdown of MLKL at 24 h before tGCI. As expected, with MLKL-siRNA treatment, the expression of TNF-α and pro-IL-1β in CA1 were significantly downregulated at 48 h after tGCI, similar to what were observed in HPC group (Fig. 2C). However, the administration of MLKL-siRNA exerted no force on the expression of IL-10 and Arg-1 (Fig. 2D).

### Up-regulation of A20 induced by HPC suppresses M1 polarization of microglia/macrophages in CA1 after tGCI

**Fig. 3:**
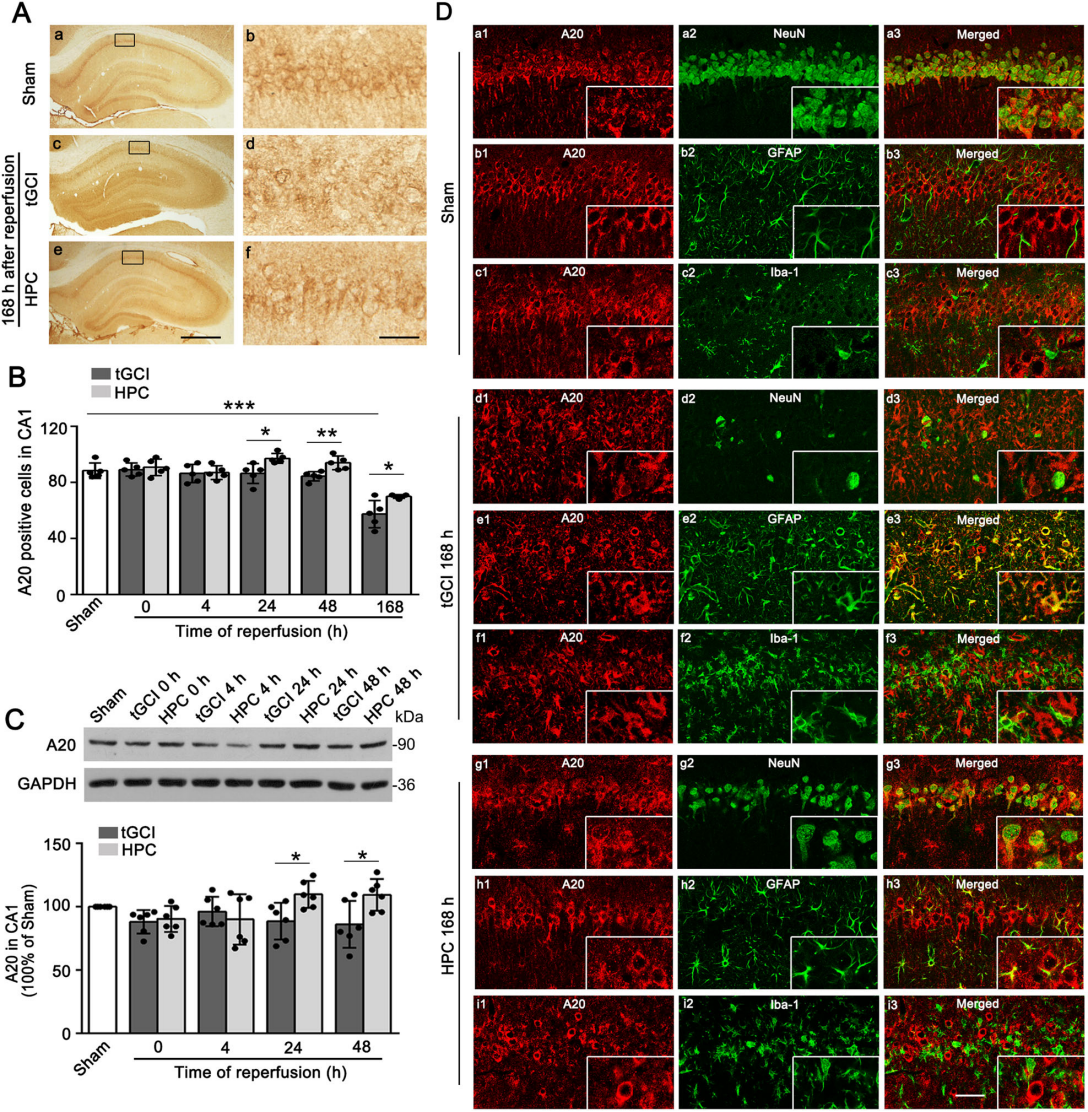
HPC upregulates the expression of A20 in CA1 after tGCI. **A** Immunohistochemistry staining of A20 in hippocampus of rats, including representative images of Sham group (a,b), and 168 h after reperfusion of tGCI group (c, d) and HPC group (e, f). Scale bar: 250 μm (a, c, e), and 25 μm (b, d, f). **B** Quantitative analysis of A20-immunoreactivities in CA1. HPC maintains the expression of A20 in CA1 at 24–168 h after tGCI. **C** Western blot analysis of A20 expression in CA1. The histogram presents the quantitative analysis of A20 in CA1. HPC maintains the level of A20 in CA1 at 24–48 h after tGCI. Each bar represents the mean ± S.D. Statistical analysis was performed using ANOVA with LSD or Tamhane’s T2 post-hoc test, or Kruskal–Wallis H test. *p < 0.05, **p < 0.01, and ***p < 0.001. **D** Representative photomicrographs with fluorescent staining of A20 (red) and NeuN/GFAP/Iba-1 (green) in CA1. HPC increases the colocalization of A20 with NeuN, and decreases the colocalization of A20 with GFAP in CA1 after tGCI. Scale bars: 75 μm.

Next, we investigated whether A20 affects tCCI-induced necroptosis and the subsequent M1 polarization of microglia/macrophages. As shown in Fig. 3A, B, A20-positive labeling was mainly located in neuron-like cells of pyramidal layer. A20-immunoreactivities largely decreased in CA1 after 168 h of tGCI, compared with Sham group. Diametrically, the reduction of A20-immunoreactivities after 168 h of tGCI could be partially prevented by HPC. Similarly, HPC maintained the expression of A20 after 24 h and 48 h of tGCI (Fig. 3C). Hypoxia without tGCI did not affect the expression of A20 in CA1 of Sham (Supplementary Fig. S1E, F). The results of double-fluorescent immunohistochemistry showed that A20 was colocalized with NeuN in Sham, indicating that A20 was predominantly expressed in neurons (Figs. 3D, a1–c3). Notably, after 168 h of tGCI, the majority of A20-positive cells turned into astrocytes, as shown in the major co-localization of A20 with GFAP (Figs. 3D, e1–e3), contrasted with only a few with NeuN (Figs. 3D, d1–d3), and none with Iba-1 (Figs. 3D, f1–f3). By contrast, A20 was expressed in a considerable number of NeuN-positive (Figs. 3D, g1–g3) and GFAP-positive (Figs. 3D, h1–h3) cells in HPC groups.

**Fig. 4:**
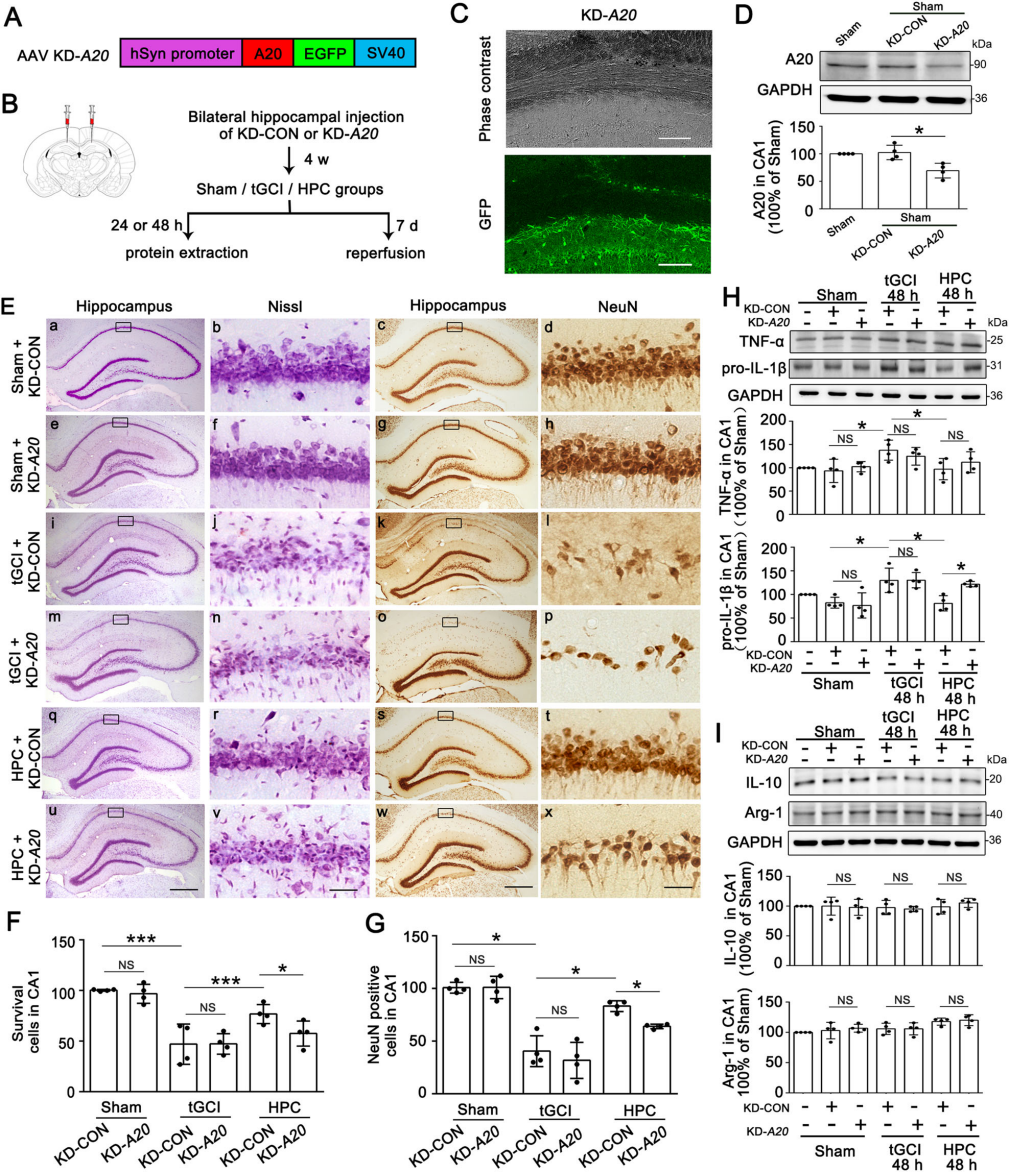
Silencing of A20 abolishes the HPC-induced neuroprotection in CA1 after tGCI. **A** The sequence of AAV vectors for silencing A20 targeting of neurons. **B** Scheme of AAV vectors injection into bilateral CA1 pyramidal layers. AAV vectors were injected into bilateral CA1 at 28 days before the surgery of four-vessel occlusion. Subsequent evaluations of AAV-injected rats were conducted at 24 h/48 h/7 d after tGCI with or without hypoxia. **C** Phase contrast and fluorescent images from coronal sections of CA1 following injection of AAV vectors. Scale bar: 75 μm. **D** Representative images of immunoblots of A20 in CA1 of Sham animals injected with KD-*A20*. The histogram presents the quantitative analysis of A20 levels. KD-*A20* significantly downregulates A20 levels in CA1 of Sham rats. **E** Representative photomicrographs of cresyl violet staining and NeuN immunostaining in CA1 from rats administered with either KD-CON or KD-*A20* at 7 d after reperfusion with or without hypoxia. Boxes indicate the magnified regions displayed in the right panel. Scale bar: 250 μm (a, c, e, g, i, k, m, o, q, s, u, w) and 25 μm (b, d, f, h, j, l, n, p,r, t, v, x). **F**, **G** Quantitative analyses of surviving cells and NeuN-positive cells in CA1. KD-*A20* decreases the number of surviving cells and NeuN-positive cells in CA1 after HPC. Each bar represents the mean ± S.D. **H**, **I** Representative images of Western blot show the expression of TNF-α, pro-IL-1β, IL-10, and Arg-1 in CA1. The histogram presents the quantitative analyses of TNF-α, pro-IL-1β, IL-10, and Arg-1. KD-*A20* upregulates the levels of pro-IL-1β in CA1 after HPC. Statistical analysis was performed using ANOVA with LSD or Tamhane’s T2 post-hoc test, or Kruskal–Wallis H test. Unpaired *t* test or Mann–Whitney *t* test was used for comparing with KD-CON group. *p < 0.05, **p < 0.01, and ***p < 0.001.NS, no significance.

As shown in the flowchart in Fig. 4A, B, AAV loaded with small-interfering RNA against A20 targeting of neurons, which resulted in knockdown of A20 (KD-*A20*) in neurons, or AAV vector carrying a scrambled control (KD-CON) was injected into bilateral CA1 at 28 days before tGCI. Fluorescent images for GFP confirmed the effective transfection of AAV into CA1 (Fig. 4C), and KD-*A20* downregulated A20 levels in CA1 of Sham rats (Fig. 4D), leading to no obvious morphological changes in neurons of Sham when compared with KD-CON treatment (Fig. 4E, a–h, F, G). Also, KD-*A20* did not affect the expression of Iba-1 and CD68 in CA1 of Sham (Supplementary Fig. S2) and did not exacerbate neuronal damage in tGCI groups (Fig. 4E, i–p, F, G). However, KD-*A20* in neurons abolished HPC-induced neuroprotection as evidenced by a significant decrease in the number of surviving cells and NeuN-positive cells (Fig. 4E, q–x, F, G). Furthermore, HPC-induced reduction of pro-IL-1β at 48 h after tGCI was also abrogated by KD-*A20* (Fig. 4H). Expectedly, KD-*A20* exerted no influence on the expression of IL-10 and Arg-1, either in tGCI or HPC rats (Fig. 4I).

**Fig. 5:**
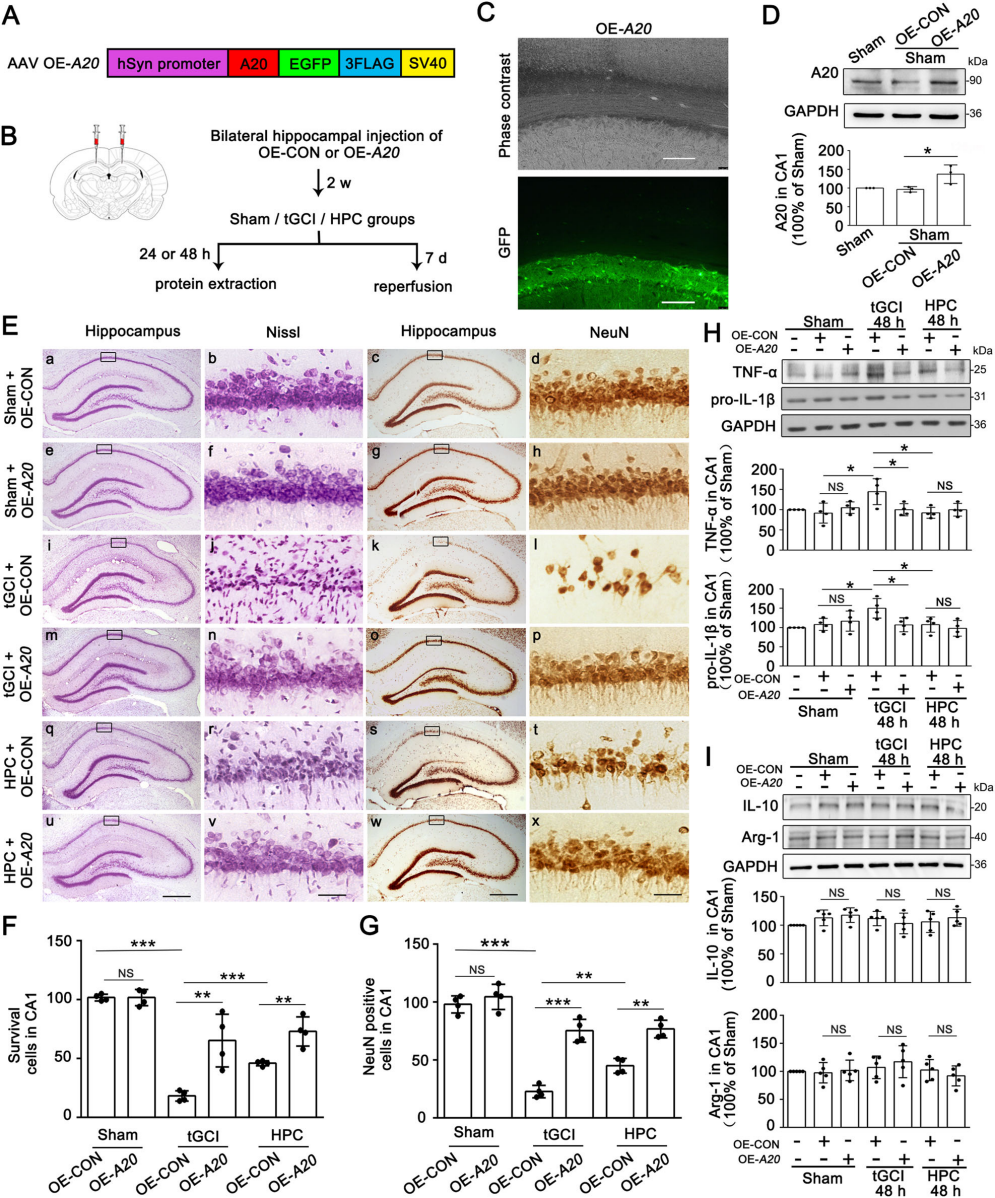
Overexpression of A20 suppresses M1 polarization of microglia/macrophages and alleviates tGCI-induced neuronal damage in CA1. **A** The sequence of AAV vectors for overexpressing A20 targeting of neurons. **B** Scheme of AAV vectors injection into bilateral CA1 pyramidal layers. AAV vectors were injected into bilateral CA1 14 d before the surgery of four-vessel occlusion. Subsequent evaluations of AAV-injected rats were conducted at 24 h/48 h/7 d after tGCI with or without hypoxia. **C** Phase contrast and fluorescent images from coronal sections of CA1 following injection of AAV vectors. Scale bar: 75 μm. **D** Representative images of immunoblots of A20 in CA1 of Sham animals injected with OE-*A20*. The histogram presents the quantitative analysis of A20 levels. OE-*A20* significantly upregulates A20 levels in CA1 of Sham rats. **E** Representative photomicrographs of cresyl violet staining and NeuN immunostaining in CA1 from rats administered with either OE-CON or OE-*A20* at 7 d after reperfusion with or without hypoxia. Boxes indicate the magnified regions displayed in the right panel. Scale bar: 250 μm (a, c, e, g, i, k, m, o, q, s, u, w) and 25 μm (b, d, f, h, j, l, n, p,r, t, v, x). **F**, **G** Quantitative analyses of surviving cells and NeuN-positive cells in CA1. OE-*A20* increases the number of surviving cells and NeuN-positive cells in CA1 after tGCI. Each bar represents the mean ± S.D. **H**, **I** Representative images of Western blot show the expression of TNF-α, pro-IL-1β, IL-10, and Arg-1 in CA1. The histogram presents the quantitative analyses of TNF-α, pro-IL-1β, IL-10, and Arg-1. OE-*A20* downregulates the levels of TNF-α and pro-IL-1β in CA1 after tGCI. Each bar represents the mean ± S.D. Statistical analysis was performed using ANOVA with LSD or Tamhane’s T2 post-hoc test, or Kruskal–Wallis H test. Unpaired *t* test or Mann–Whitney *t* test was used for comparing with OE-CON group. *p < 0.05, **p < 0.01, and ***p < 0.001. NS, no significance.

As shown in the flowchart in Fig. 5A, B, A20-AAV vectors, which resulted in overexpression of A20 (OE-*A20*) in neurons, or the negative control vectors (OE-control, OE-CON), were injected into bilateral CA1 14 d before tGCI. The effectiveness of AAV transfection into CA1 was verified by fluorescence for GFP (Fig. 5C). In CA1 of Sham, the expression of A20 was elevated after OE-*A20* administration (Fig. 5D), but neither OE-*A20* nor OE-CON altered the morphology and the number of surviving or NeuN-positive cells (Fig. 5E, a–h, F, G). Also, OE-*A20* did not affect the expression of Iba-1 and CD68 in CA1 of Sham (Supplementary Fig. S3). Notably, OE-*A20* markedly ameliorated neuronal loss compared with OE-CON after tGCI (Fig. 5E, i–p, F, G). Additive neuroprotective effect was observed after OE-*A20* and HPC were both applied (Fig. 5E, q–x, F, G). The administration of OE-*A20* reduced the expression of TNF-α and pro-IL-1β at 48 h after tGCI, consistent with the observations in HPC rats (Fig. 5H). Unsurprisingly, the injection of OE-*A20* did not disturb the expression of IL-10 and Arg-1 after tGCI with or without hypoxia (Fig. 5I).

### Up-regulation of A20 induced by HPC negatively regulates necroptosis through restricting the ubiquitination of RIP3 in CA1 after tGCI

**Fig. 6:**
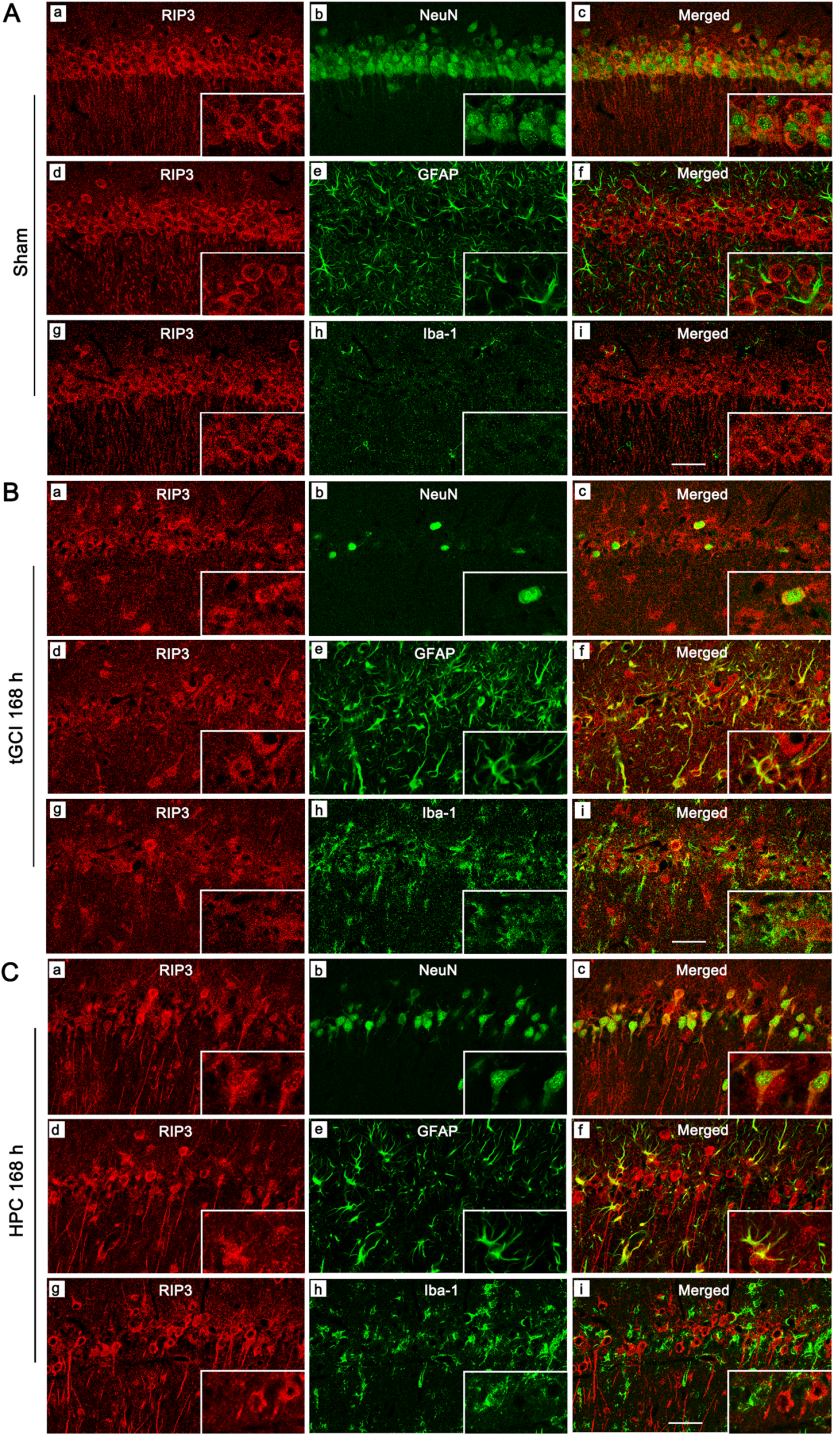
The effects of HPC on the localization of RIP3 in CA1 after tGCI. Representative photomicrographs with fluorescent staining of RIP3 (red) and NeuN/GFAP/Iba-1 (green) in CA1 of Sham group (**A**), tGCI 168 h group (**B**), and HPC 168 h group (**C**). HPC increases the colocalization of RIP3 with NeuN, and decreases the colocalization of RIP3 with GFAP in CA1 after tGCI. Scale bar: 75 µm.

**Fig. 7:**
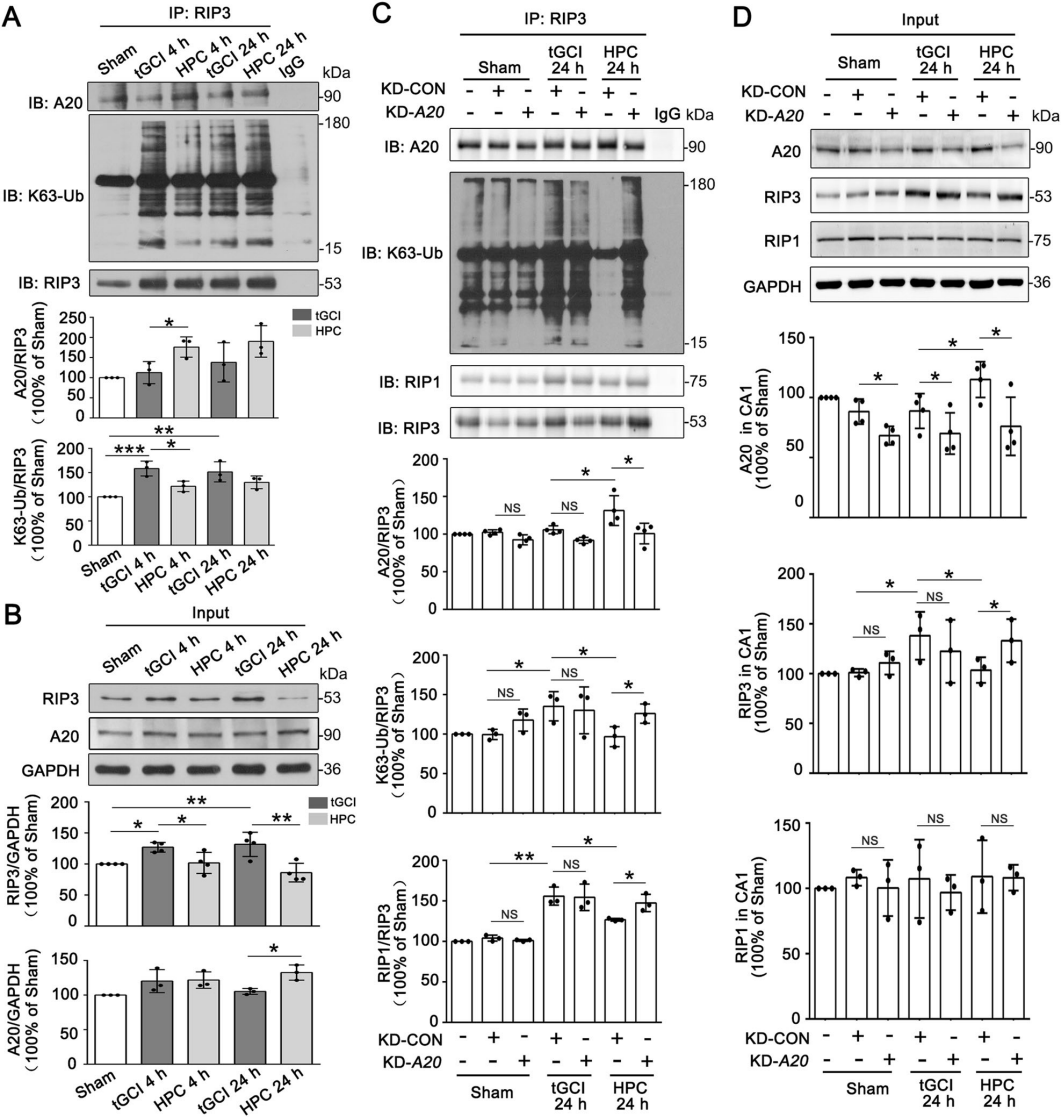
HPC or silencing of A20 disrupts the interaction between A20 and RIP3, and promotes K63-linked ubiquitination of RIP3 in CA1 after tGCI. **A** Immunoprecipitation assays show the interaction between RIP3 and A20, and K63-linked ubiquitination of RIP3 in CA1 of tGCI and HPC groups. HPC enhances the interaction of A20-RIP3, and diminishes K63-linked ubiquitination of RIP3 in CA1 at 4 h after tGCI. **B** Representative images of Western blot show the expression of RIP3 and A20 in CA1 of tGCI and HPC groups. HPC suppresses the expression of RIP3 after tGCI. Each bar represents the mean ± S.D. Statistical analysis was performed using ANOVA with LSD or Tamhane’s T2 post-hoc test, or Kruskal–Wallis H test. *p < 0.05, **p < 0.01, and ***p < 0.001. **C** Immunoprecipitation assays show the effects of KD-*A20* on the interaction between RIP3 and A20, K63-linked ubiquitination of RIP3, and the formation of RIP1-RIP3 complex in CA1 after tGCI with or without HPC. KD-*A20* disrupts the interaction between A20 and RIP3, elevates K63-linked ubiquitination of RIP3, and enhances the interaction between RIP3 and RIP1 in CA1 of HPC rats. **D** Immunoblots assays show the effects of KD-*A20* on the expression of A20, RIP3 and RIP1 in CA1 after tGCI with or without HPC. KD-*A20* increases the expression of RIP3 in CA1 of HPC rats. Each bar represents the mean ± S.D. Statistical analysis was performed using ANOVA with LSD or Tamhane’s T2 post-hoc test, or Kruskal–Wallis H test. Unpaired *t* test or Mann–Whitney *t* test was used for comparing with KD-CON group. *p < 0.05, **p < 0.01, and ***p < 0.001. NS, no significance.

Double immunostaining showed that RIP3-positive cells in CA1 of Sham were mainly expressed on NeuN-positive cells (Fig. 6A, a–c), rather than on GFAP- (Fig. 6A, d–f) or Iba-1-positive cells (Fig. 6B, g–i), indicating that RIP3 was predominantly localized in neurons of Sham. In particular, after 168 h of tGCI, most of the RIP3-positive cells were colocalized with GFAP (Fig. 6B, d–f), rather than with NeuN (Fig. 6B, a–c) or Iba-1 (Fig. 6B, g–i), suggesting that RIP3 was predominantly localized in astrocytes after tGCI. Alternatively, RIP3 was colocalized within both NeuN-positive (Fig. 6C, a–c) and GFAP-positive cells (Fig. 6C, d–f) in HPC rats, rather than in Iba-1-positive cells (Fig. 6C, g–i). These results imply existing similarities in the cellular localization between RIP3 and A20 in CA1. We hence further checked the interaction between A20 and RIP3 (Fig. 7A). Compared with tGCI groups, an enhancement in the interaction of A20-RIP3 was observed at 4 h of reperfusion in HPC rats. Given the deubiquitinating enzyme activity of A20, we wondered whether the interaction of A20-RIP3 contributed to the ubiquitination of RIP3. As expected, tGCI rats exhibited increased levels of K63-linked ubiquitination of RIP3, whereas HPC significantly diminished K63-linked ubiquitination of RIP3 at 4 h of reperfusion. Consistent with our previous studies, the expression of RIP3 was markedly elevated in tGCI rats at 4 h and 24 h after reperfusion, but was significantly suppressed in HPC rats at 24 h (Fig. 7B).

**Fig. 8:**
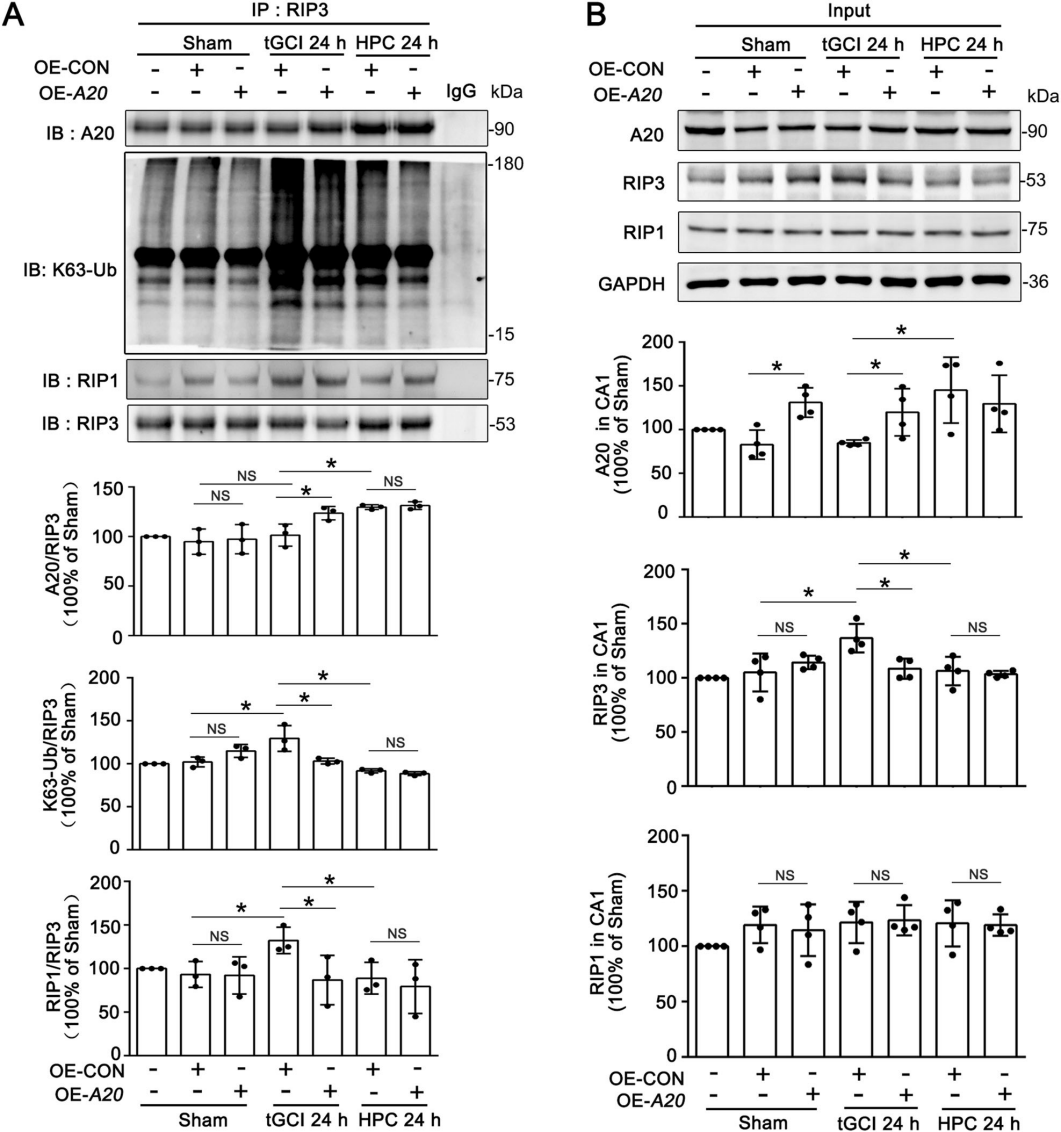
Overexpression of A20 promotes the interaction between A20 and RIP3, and decreased K63-linked ubiquitination of RIP3 in CA1 after tGCI. **A** Immunoprecipitation assays show the effects of OE-*A20* on the interaction between RIP3 and A20, K63-linked ubiquitination of RIP3, and the formation of RIP1-RIP3 complex in CA1 after tGCI with or without HPC. OE-*A20* promotes the interaction between A20 and RIP3, decreases K63-linked ubiquitination of RIP3, and inhibits the interaction between RIP1 and RIP3 in CA1 after tGCI. **B** Immunoblots assays show the effects of OE-*A20* on the expression of A20, RIP3 and RIP1 in CA1 after tGCI with or without HPC. OE-*A20* decreases the expression of RIP3 in CA1 after tGCI. Each bar represents the mean ± S.D. Statistical analysis was performed using ANOVA with LSD or Tamhane’s T2 post-hoc test, or Kruskal–Wallis H test. Unpaired *t* test or Mann–Whitney *t* test was used for comparing with OE-CON group. *p < 0.05, **p < 0.01, and ***p < 0.001. NS, no significance.

In consequence, we assume that K63-linked ubiquitination of RIP3 is essential for A20 to regulate necroptosis. Thus, AVV-mediated knockdown or overexpression of A20 in neurons was performed in CA1 of rats. As shown in Fig. 7C, D, KD-*A20* disrupted the interaction between A20 and RIP3 and elevated K63-linked ubiquitination of RIP3 in HPC rats. Remarkably, KD-*A20* not only increased the expression of RIP3 but also enhanced the interaction between RIP3 and RIP1. However, KD-*A20* did not impact the expression of RIP1. In contrast, compared with the OE-CON tGCI group, OE-*A20* promoted the interaction between A20 and RIP3, and decreased K63-linked ubiquitination of RIP3, accompanied with the reduction of interaction between RIP1 and RIP3 at 24 h of tGCI (Fig. 8A). In addition, OE-*A20* decreased the expression of RIP3 after tGCI, but did not disturb the expression of RIP1 (Fig. 8B). Nevertheless, OE-*A20* did not affect the interactions between A20 and RIP3, between RIP1 and RIP3 neither in Sham nor in HPC rats. Also, OE-*A20* in Sham or HPC groups did not affect the expression and the K63-linked ubiquitination of RIP3 in CA1.

## Discussion

In this study, we found activation of microglia/macrophages in CA1 at 168 h after tGCI. Nonetheless, microglia/macrophages in HPC rats displayed a more resting morphology. These results imply that HPC inhibits the activation of microglia/macrophages in CA1 after tGCI. Activated microglia/macrophages can polarize into two rather different phenotypes. The previous studies showed a dominance of M2 phenotype microglia in the initial hours after cerebral ischemia, which gradually shifted to M1 phenotype [8]. In the present study, we found that both iNOS- and CD206-positive microglia/macrophages increased after tGCI, which suggests that both M1 and M2 phenotype microglia/macrophages were activated after tGCI. This phenomenon was further proven by a sharp increase in mRNA levels of TNF-α, IL-1β, IL-10 and Arg-1 after tGCI. However, in stark contrast to the observed results of immunofluorescence and qRT-PCR, we found that the protein expression levels of the M2 phenotype-associated inflammatory molecules IL-10 and Arg-1 have barely changed after tGCI, which suggest increased M1 phenotype in CA1 after tGCI. We further demonstrated that HPC reduced the number of iNOS-positive microglia/macrophages and downregulated the mRNA and protein levels of TNF-α and IL-1β after tGCI. Collectively, we propose that HPC inhibits the activation of M1 microglia/macrophages in CA1 after tGCI, thereby alleviating the inflammatory response.

Recently, necroptosis has been implicated in the regulation of inflammatory responses and microglial polarization [37]. Especially, Yang et al. reported that focal cerebral ischemia-induced RIP3/MLKL-mediated neuronal necroptosis, and depleting RIP3 or MLKL ameliorated inflammatory responses induced by cerebral ischemia/reperfusion [19]. Besides, knockout of RIP3 or MLKL switched activated microglia toward M2 type in the ischemic cortex [19]. We recently reported HPC inhibited the activation of MLKL, and thus protecting CA1 neurons from tGCI-induced damage [15]. In this study, we observed that treatment with an inhibitor of necroptosis, Nec-1 or MLKL siRNA, significantly reduced the production of pro-inflammatory cytokines in CA1 after tGCI and this also prevented microglial polarization towards M1 type, which could be mimicked by HPC. Taken together, these data indicate that HPC alleviates necroptosis in CA1 after tGCI, thereby inhibiting the M1 polarization of microglia/macrophages.

The function of RIP3 in microglia/macrophages has been explored in the previous studies [38, 39]. However, in our study, RIP3 is rarely expressed in microglia of CA1, but predominantly localized in neurons of Sham. Under physiological conditions, the hippocampal CA1 pyramidal cell layer are mainly composed of a large number of neurons and a very small amount of resting glial cells. Therefore, the number of RIP3-positive microglia could be underestimated due to the massive presence of RIP3-positive neurons in the same areas. In previous work, we demonstrated that neuronal necroptosis, featured by activation and interaction of RIP3 and MLKL, occurred at the early stage of reperfusion after tGCI [14]. However, phosphorylated MLKL was mainly distributed in microglia [15], whereas RIP3 in astrocytes, at 168 h after tGCI of reperfusion. Similar results were obtained in a previous study showing neuronal necroptosis occurred at the early stage whereas astrocyte necroptosis at the late stage after focal cortical ischemia in mice [19]. RIP3 acts as a vital upstream factor for phosphorylation of MLKL and an indispensable part of the necrosome complex, it seems that phosphorylated MLKL and necrosomes cannot be formed in microglia. Therefore, we speculate that necroptotic neurons and astrocytes, as well as the cellular contents released from those, play a crucial role in the activation of M1 microglia/macrophages after tGCI.

The molecular mechanisms by which inhibition of necroptosis mediated by HPC prevents M1 polarization of microglia/macrophages and protects against cerebral ischemic injury remain largely unexplored. Recent studies have identified A20 as a novel regulator of necroptosis after intracerebral hemorrhage [34] and traumatic brain injury [33]. Moreover, A20 deficient T cells and fibroblasts are vulnerable to necroptosis [25]. Additionally, recent studies provide some new evidences of A20 in regulating neuroinflammation [40, 41]. To clarify the effect of A20 on necroptosis and polarization of microglia/macrophages, we constructed AAV targeting silencing or overexpressing neuronal A20, respectively. The results demonstrated that selective silence of A20 in neurons not only promoted the interaction between RIP1 and RIP3, abolished HPC-induced neuroprotection against tGCI, activated M1 microglia/macrophages and also finally exacerbated inflammatory responses, whereas overexpression of A20 exerted the opposite effects. In short, our study, focusing on the regulation of necroptosis by A20 in neurons, suggests that A20 contributed to the neuroprotective role of HPC, via alleviation of neuronal necroptosis and inhibition of M1 microglia/macrophages.

The function of A20 was shown to be mainly dependent on its capacity to regulate ubiquitin-dependent signaling cascades [42]. A20 proteins are known to contain a zinc finger domain at the carboxy terminus and an ovarian tumor domain at the amino terminus, which provide with the dual functions of ubiquitination and deubiquitination. A20 modified the polyubiquitination of substrates through ubiquitin editing and participated in apoptosis and inflammation [26, 43, 44]. Intriguingly, A20 has also been identified as a deubiquinase to directly remove K63-polyubiquitin from RIP3 and then inhibited RIP1-RIP3 necrosome formation [25, 45]. Consistent with these observations, our results showed that either HPC or overexpression of A20 in neurons could enhance the interaction of A20 and RIP3, reduce the level of K63-ubiquitination of RIP3, accompanied with the reduction of interaction between RIP1 and RIP3, and ultimately alleviate neuronal death in CA1 after tGCI, suggesting that A20-mediated deubiquitination was involved in neuroprotection of HPC against tGCI-induced necroptosis. Notably, the knockdown of A20 not only elevated K63-linked ubiquitination of RIP3 but also increased the expression of RIP3 in CA1 of HPC rats, indicating that A20 reduces the K63-linked polyubiquitination and the expression of RIP3. However, what remains unknown is whether the reduction in the level of RIP3 by A20 is caused by the decreased K63-linked ubiquitination of RIP3. It is known that K48-linked ubiquitylation targets RIP1 or RIP3 for their degradation [46]. However, there have been no reports about whether A20 also restricts RIP3-mediated necroptosis by targeting RIP3 for proteasomal degradation, which is dependent on its ubiquitin ligase activity. Further investigation, such as mutations to the A20 ZnF7 Ub-binding surface, will be required to elucidate the mechanism underlying the regulation of the expression of RIP3 by A20.

It is known that A20 is a key endogenous inhibitor of inflammatory cytokines and expresses in microglia/macrophages [29, 41]. However, in our study, we did not observe the A20 expression in microglia of CA1 after tGCI with or without hypoxia. Besides microglial cells, astrocytes have the ability to produce inflammatory cytokines [47]. Therefore, A20 expressed in astrocytes may regulate the release of inflammatory cytokines from astrocytes [48]. In addition, the intercellular crosstalk also participates in the regulation of the expression of inflammatory cytokines [49, 50]. Taken together, although not expressed in microglia, A20 plays a key role in the regulation of inflammatory cytokines in CA1 after tGCI with HPC.

In summary, our study demonstrates that HPC inhibits the activation of M1 microglia/macrophages and attenuates the inflammatory response in CA1 after tGCI. Moreover, HPC plays its neuroprotective role by upregulating the expression of A20, thereby inhibiting the K63-ubiquitination of RIP3 and alleviating neuronal necroptosis. To the best of our knowledge, this is the first time the association between microglia/macrophages polarization and necroptosis after tGCI has been documented, which depends on A20-regulated ubiquitination of RIP3. These results reveal a crosstalk between neuronal necroptosis and microglia/macrophages polarization, demonstrating a novel mechanism of cerebral ischemia tolerance mediated by HPC.

## Supplementary information

Supplemental figures

Supplemental Material & cited in the manuscript-uncropped Western blots

## Data Availability

Data will be made available on request.
