# Supplementary material for: A20 negatively regulates necroptosis-induced microglia/macrophages polarization and mediates cerebral ischemic tolerance via inhibiting the ubiquitination of RIP3
Source: Cell Death Dis. 2024 Dec 18;15(12):904. doi: 10.1038/s41419-024-07293-2 (PMC11655947; doi:10.1038/s41419-024-07293-2)
Supplement: Supplementary file 1 — Supplemental figures [file 41419_2024_7293_MOESM1_ESM.doc]

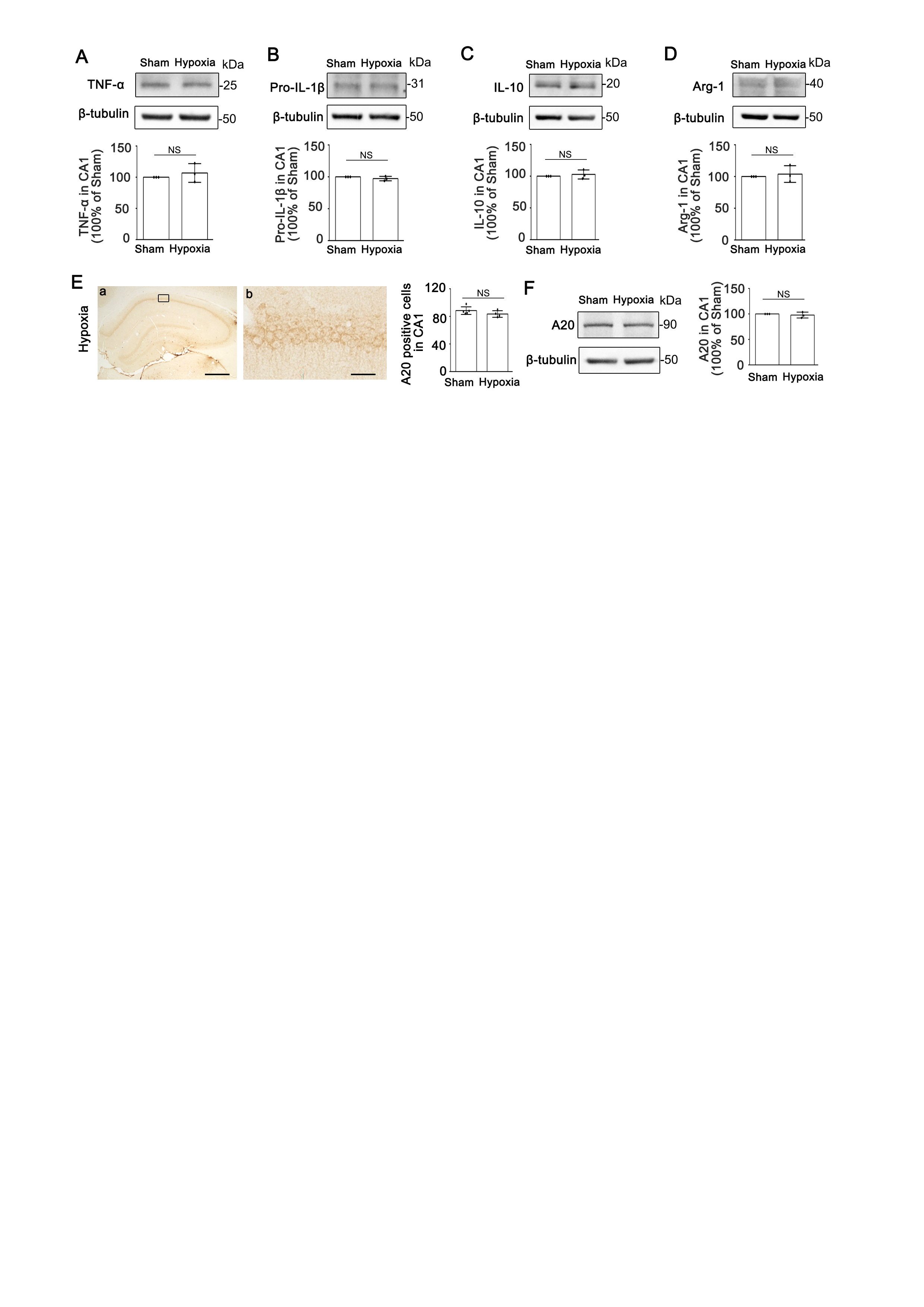


**Fig. S1 The effects of HPC on the polarization of microglia/macrophages and the expression of A20 in CA1.** Representative images of Western blot show the expression of TNF-α **(A)**, pro-IL-1β **(B)**, IL-10 **(C)**, and Arg-1 **(D)** in CA1 with or without hypoxia. The histogram presents the quantitative analyses of TNF-α, pro-IL-1β, IL-10, and Arg-1 in CA1. **(E)** Immunohistochemistry staining of A20 in hippocampus of rats (a), including representative images in hippocampal CA1 area of Hypoxia group (b). Scale bar: 250 μm (a), and 25 μm (b). The histogram presents the quantitative analysis of A20-positive cells counts in CA1. **(F)** Western blot analysis of A20 expression in CA1 with or without hypoxia. The histogram presents the quantitative analysis of A20 expression levels in CA1. Each histogram bar represents the mean±S.D. Statistical analysis was performed using Unpaired t test. NS, no significance.


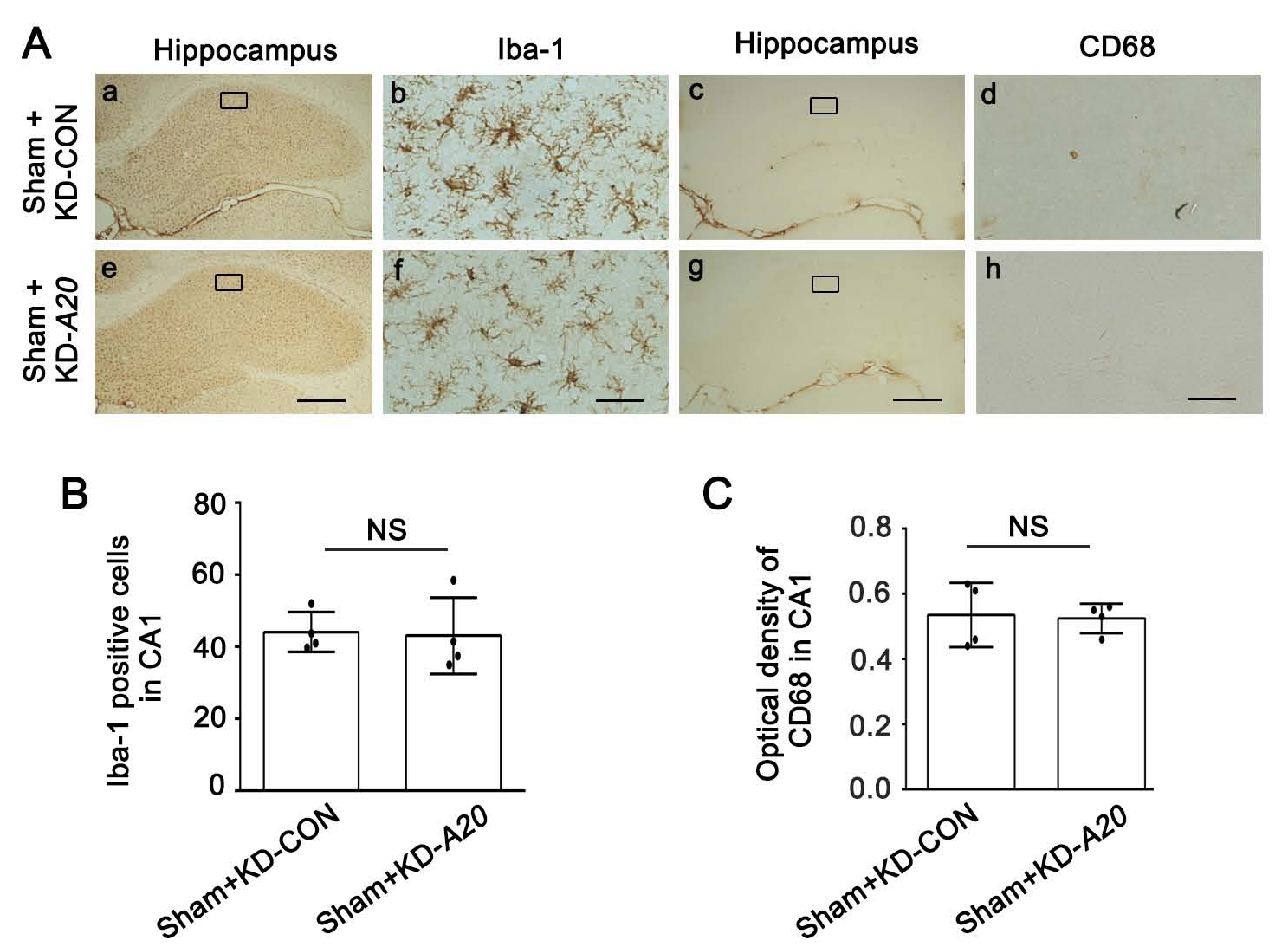


**Fig. S2 The effects of A20 knockdown on the polarization of microglia/macrophages in CA1. (A)** Representative microphotographs of Iba-1 (a, b, e, f) and CD68 (c, d, g, h) immunostaining in CA1 from rats, when administrated bilaterally with KD-CON or KD-*A20*. Scale bar: 250 μm (a, c, e, g) and 25 μm (b, d, f, h). The histograms present quantitative analyses of Iba-1-positive cell counts **(B)** and optical density of CD68-immunoreactivities **(C)** in CA1. Each bar represents the mean±S.D. Statistical analysis was performed using Unpaired t test. NS, no significance.


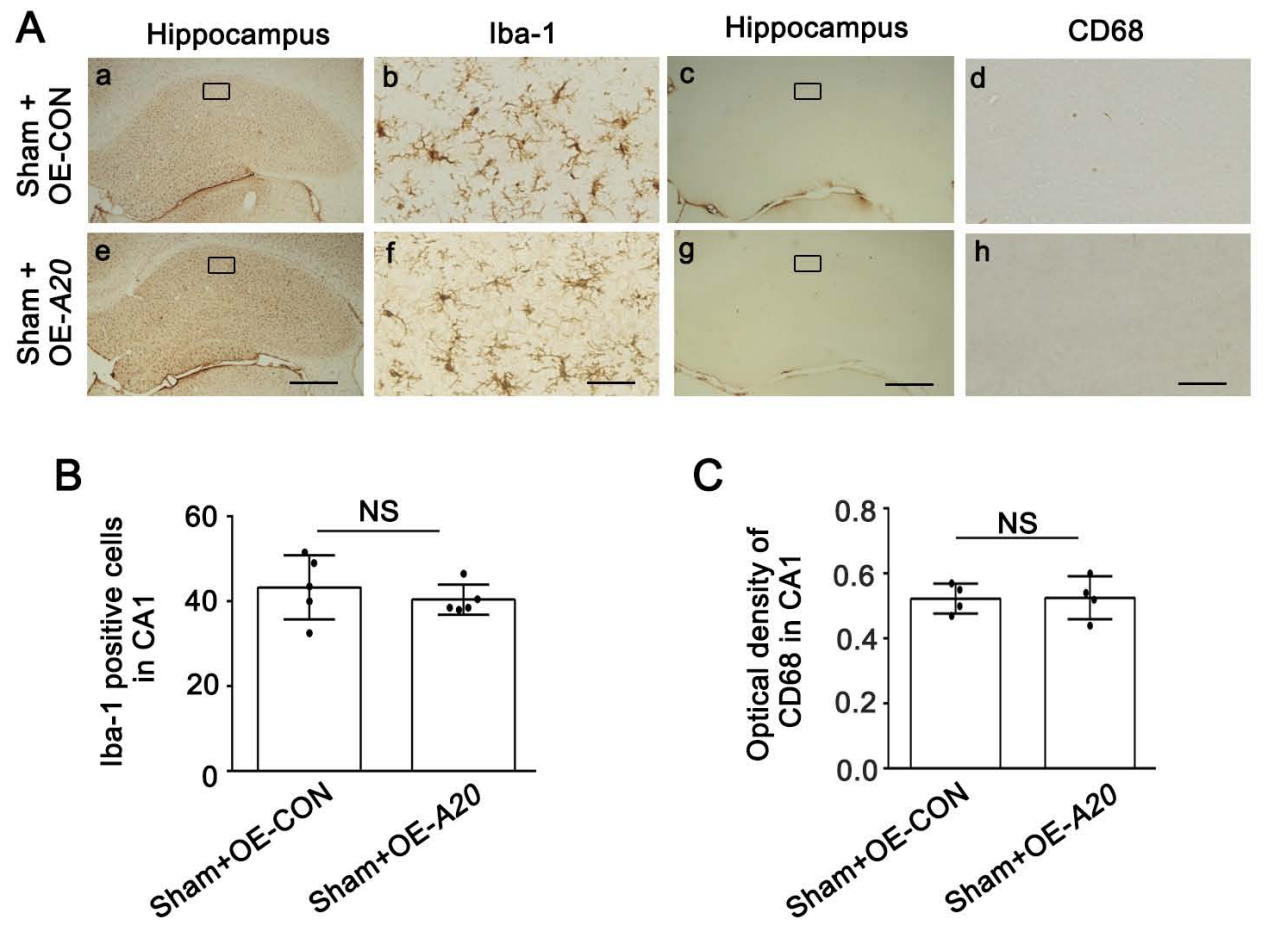


**Fig. S3** **The effects of A20 overexpression on the polarization of microglia/macrophages in CA1.** **(A)** Representative microphotographs of Iba-1 (a, b, e, f) and CD68 (c, d, g, h) immunostaining in CA1 from rats, when administrated bilaterally with OE-CON or OE-*A20*. Scale bar: 250 μm (a, c, e, g) and 25 μm (b, d, f, h). The histograms present quantitative analyses of Iba-1-positive cell counts **(B)** and optical density of CD68-immunoreactivities **(C)** in CA1. Each bar represents the mean±S.D. Statistical analysis was performed using Unpaired t test. NS, no significance.
