# Supplementary material for: A20 negatively regulates necroptosis-induced microglia/macrophages polarization and mediates cerebral ischemic tolerance via inhibiting the ubiquitination of RIP3
Source: Cell Death Dis. 2024 Dec 18;15(12):904. doi: 10.1038/s41419-024-07293-2 (PMC11655947; doi:10.1038/s41419-024-07293-2)
Supplement: Supplementary file 2 — Supplemental Material & cited in the manuscript-uncropped Western blots [file 41419_2024_7293_MOESM2_ESM.pdf]

Fig.1J

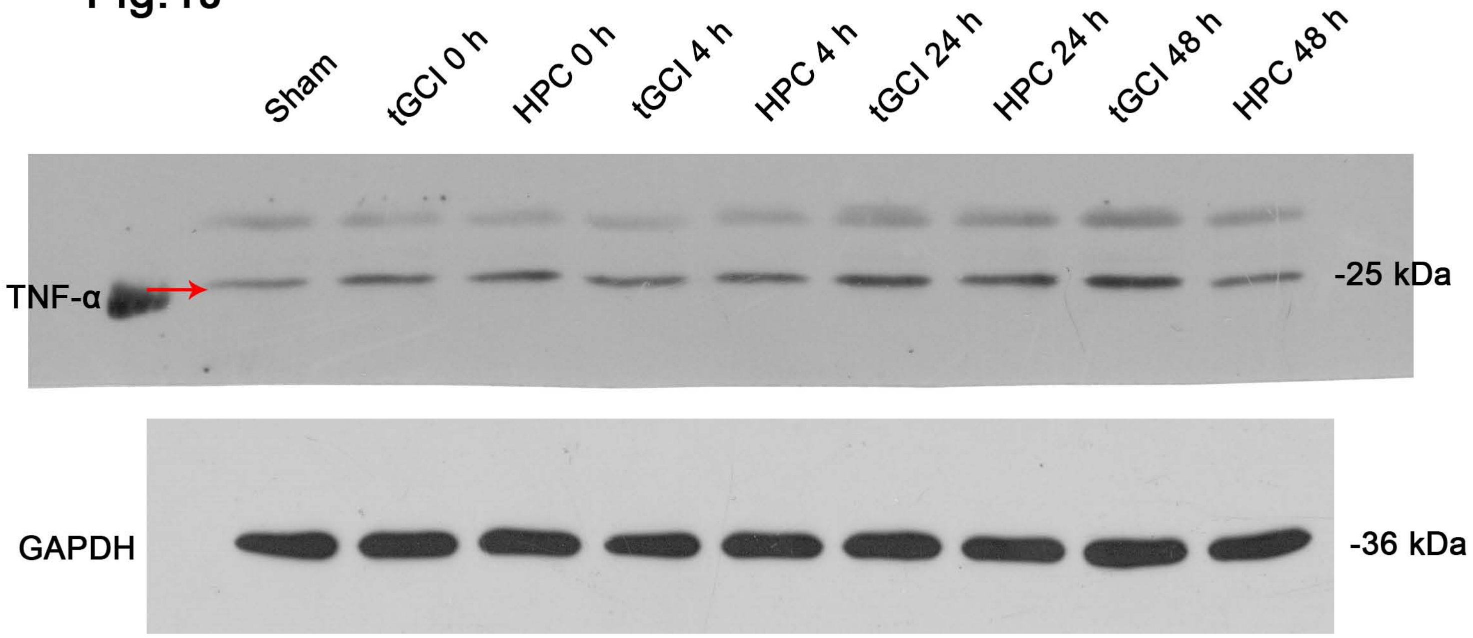
Sham
tGCI 0 h
HPC 0 h
tGCI 4 h
HPC 4 h
tGCI 24 h
HPC 24 h
tGCI 48 h
HPC 48 h
TNF-α
-25 kDa
GAPDH
-36 kDa

Fig.1K

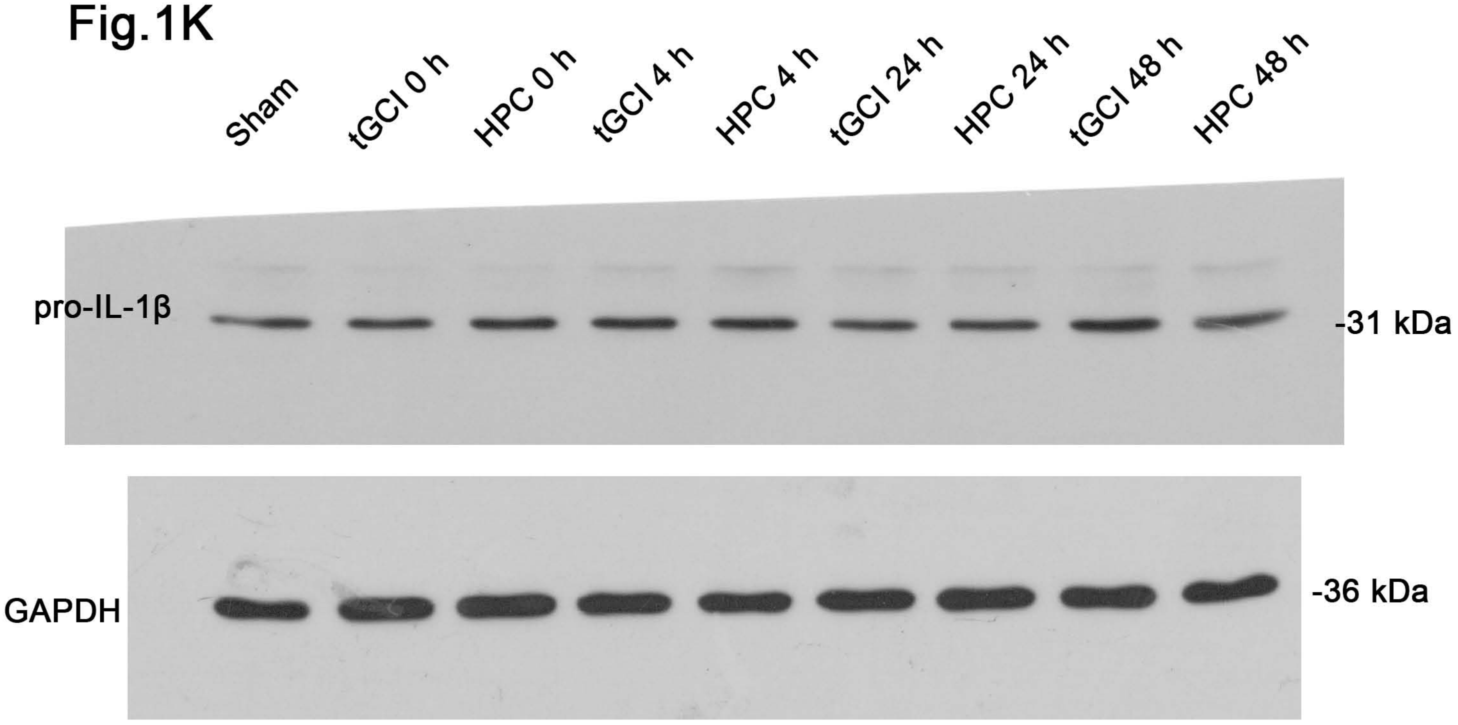
Sham
tGCI 0 h
HPC 0 h
tGCI 4 h
HPC 4 h
tGCI 24 h
HPC 24 h
tGCI 48 h
HPC 48 h
pro-IL-1β
-31 kDa
GAPDH
-36 kDa

Fig.1L

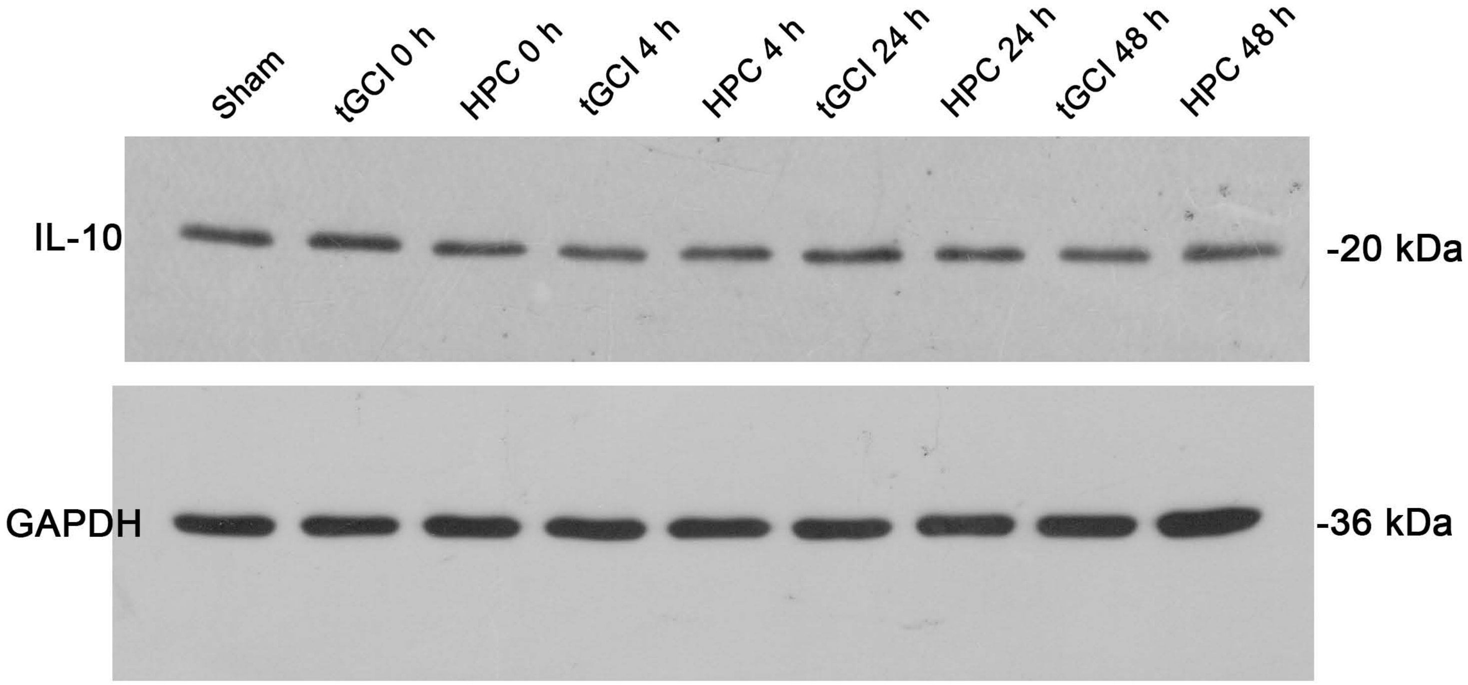

Sham
tGCI 0 h
HPC 0 h
tGCI 4 h
HPC 4 h
tGCI 24 h
HPC 24 h
tGCI 48 h
HPC 48 h
IL-10
-20 kDa
GAPDH
-36 kDa

Fig.1M

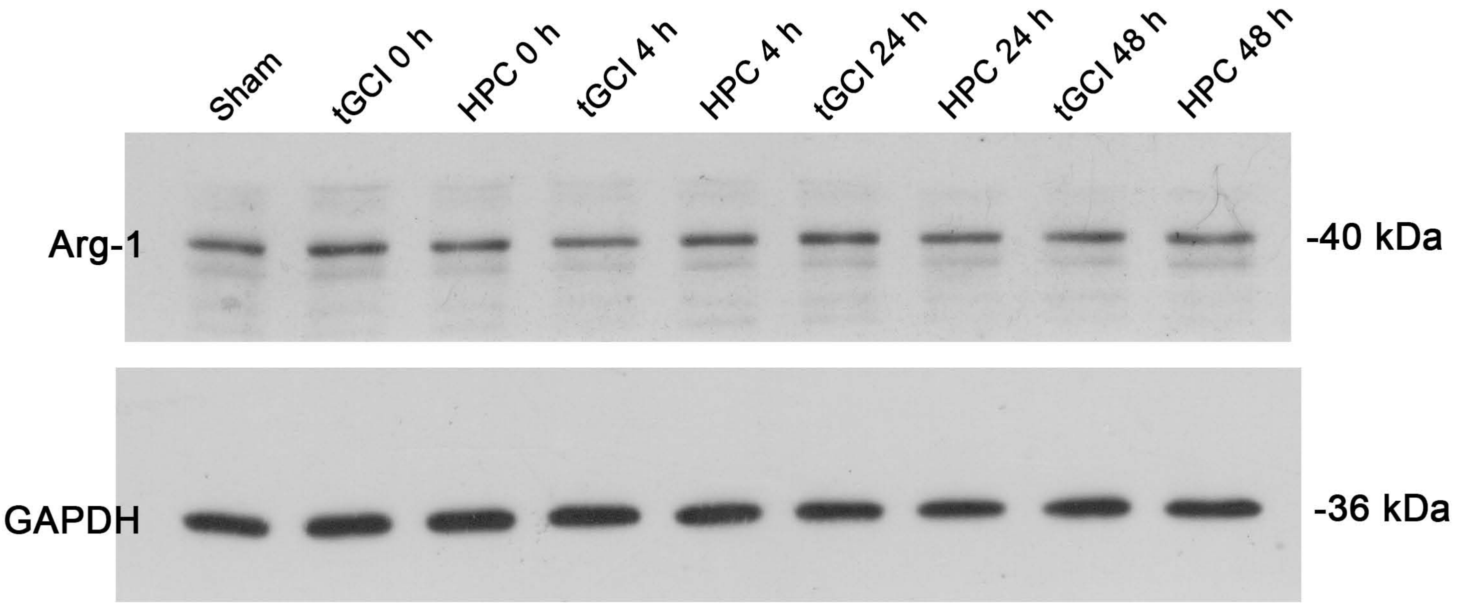

Sham
tGCI 0 h
HPC 0 h
tGCI 4 h
HPC 4 h
tGCI 24 h
HPC 24 h
tGCI 48 h
HPC 48 h
Arg-1
-40 kDa
GAPDH
-36 kDa

Fig.2A

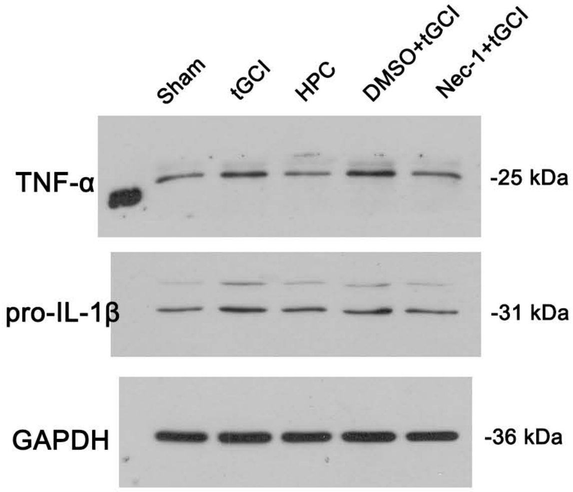

Fig.2C

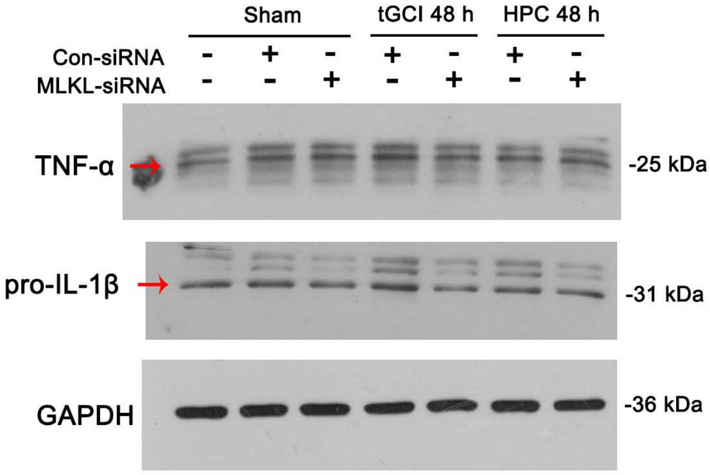

Fig.2B

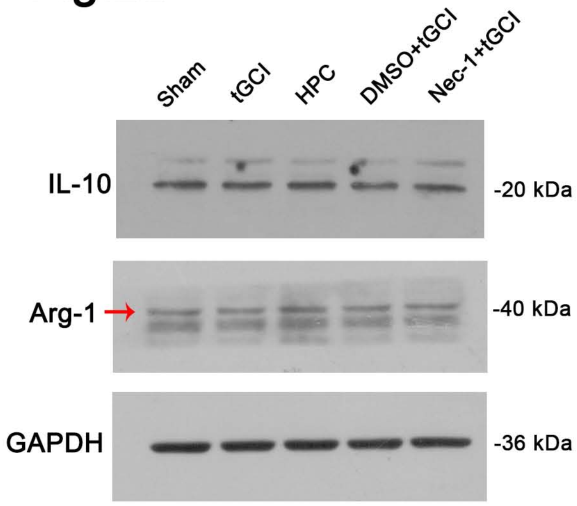

Fig.2D

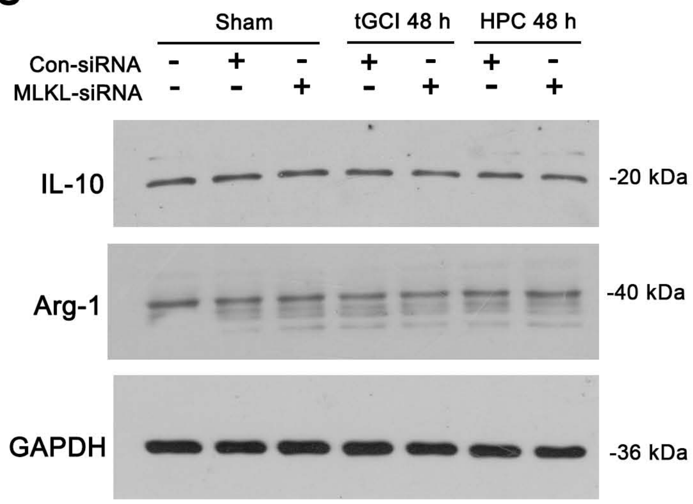

Fig.3C

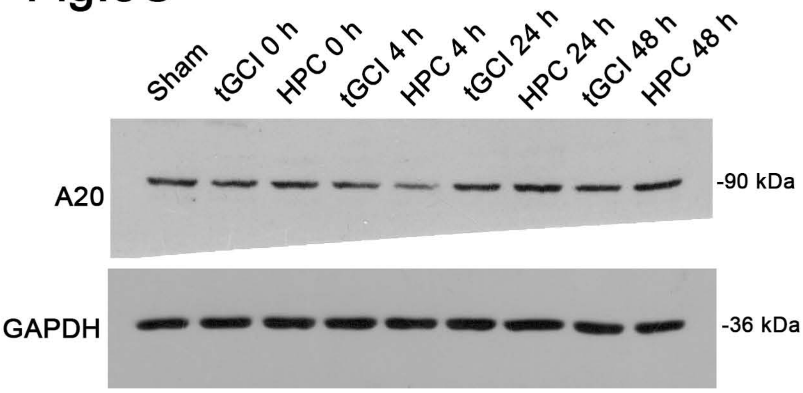

Fig.4D

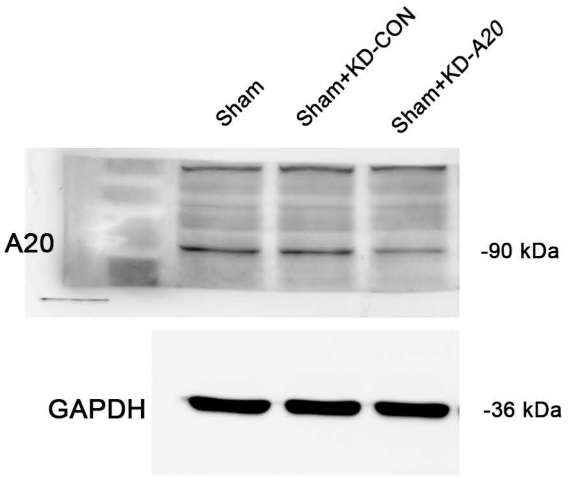

Fig.4H

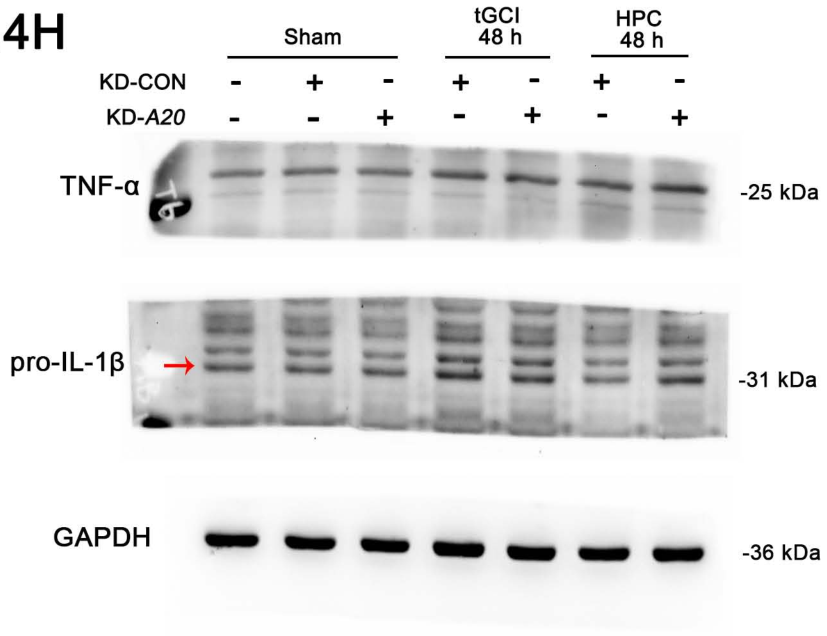

Fig. 4I

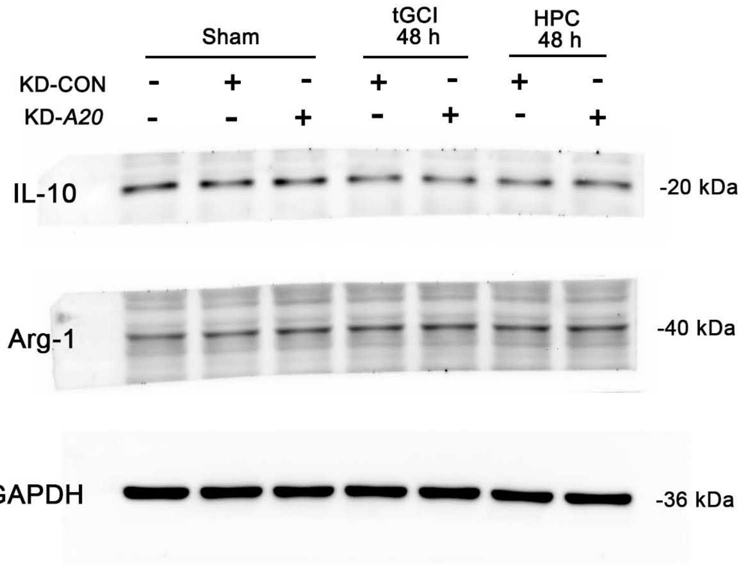

Fig.5D

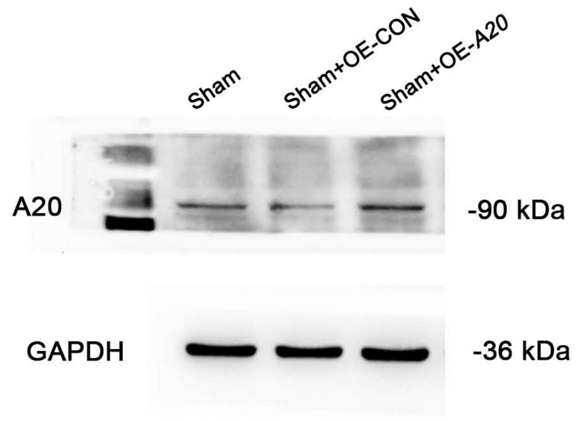

Fig.5H

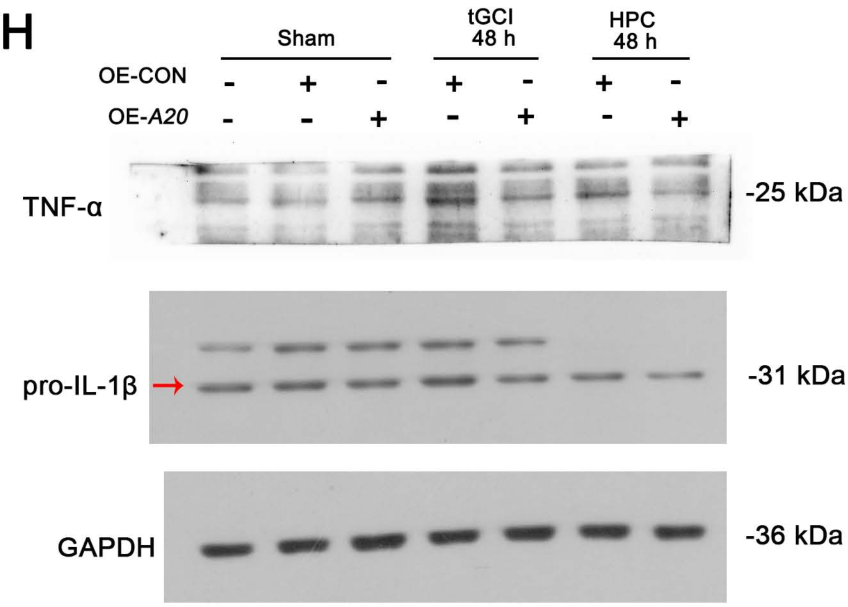

Fig. 5I

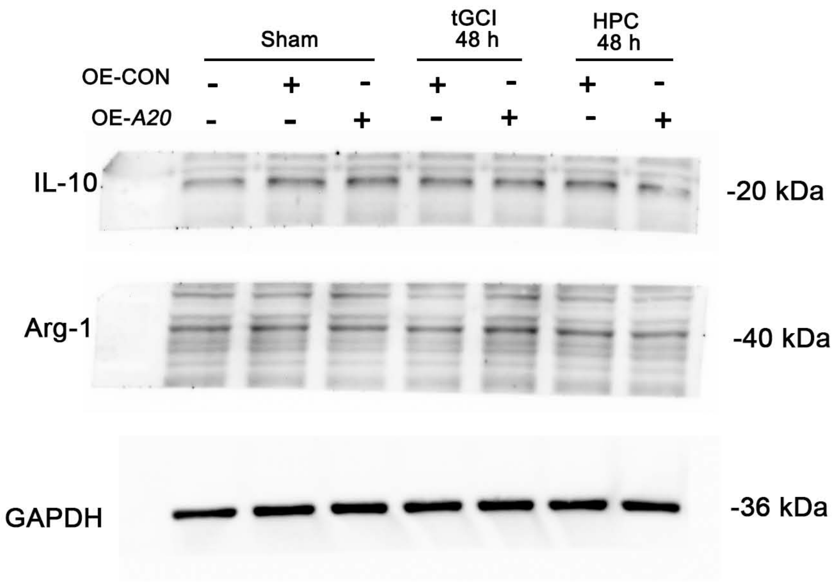

Fig.7A

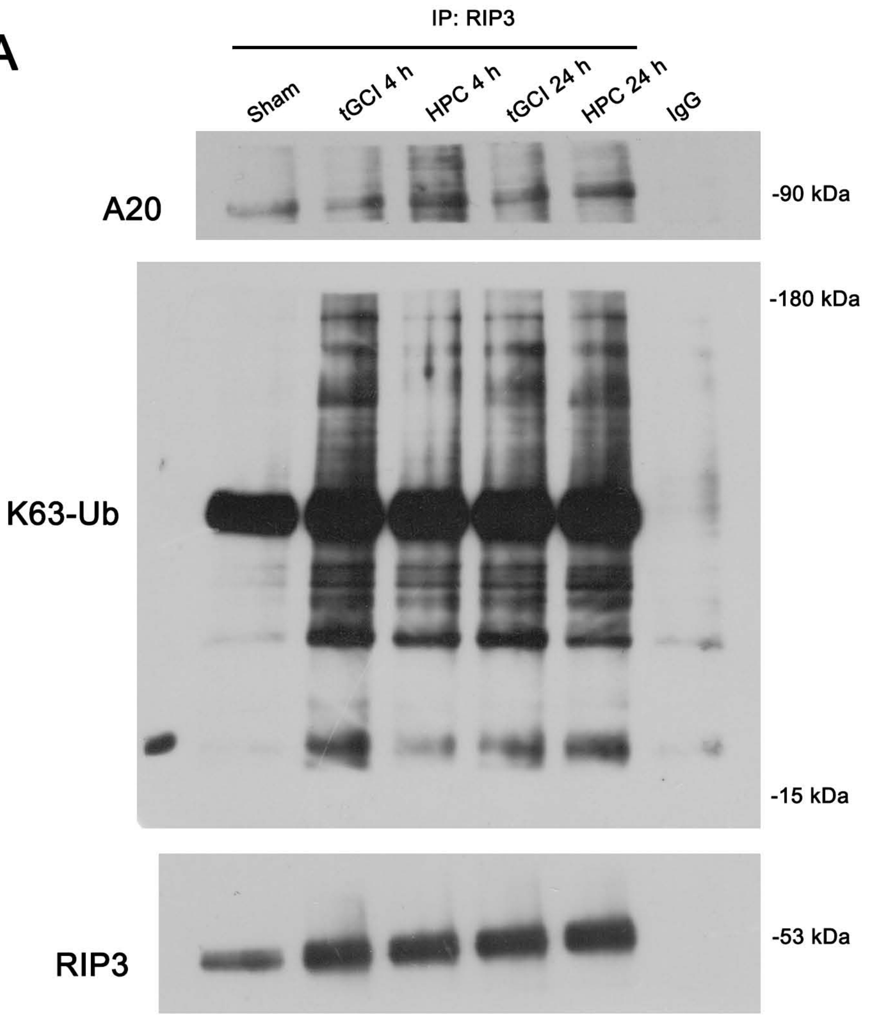
IP: RIP3
Sham
tGCI 4 h
HPC 4 h
tGCI 24 h
HPC 24 h
IgG
A20
-90 kDa
K63-Ub
-180 kDa
-15 kDa
RIP3
-53 kDa

Fig.7B

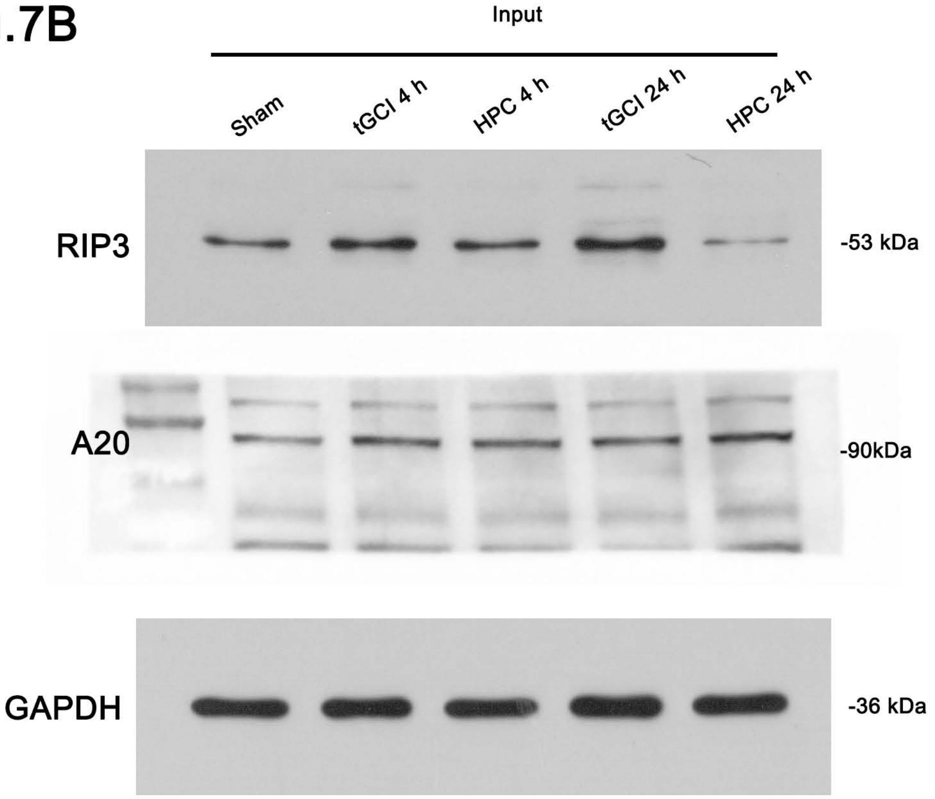
Input
Sham
tGCI 4 h
HPC 4 h
tGCI 24 h
HPC 24 h
RIP3
-53 kDa
A20
-90kDa
GAPDH
-36 kDa

Fig.7C

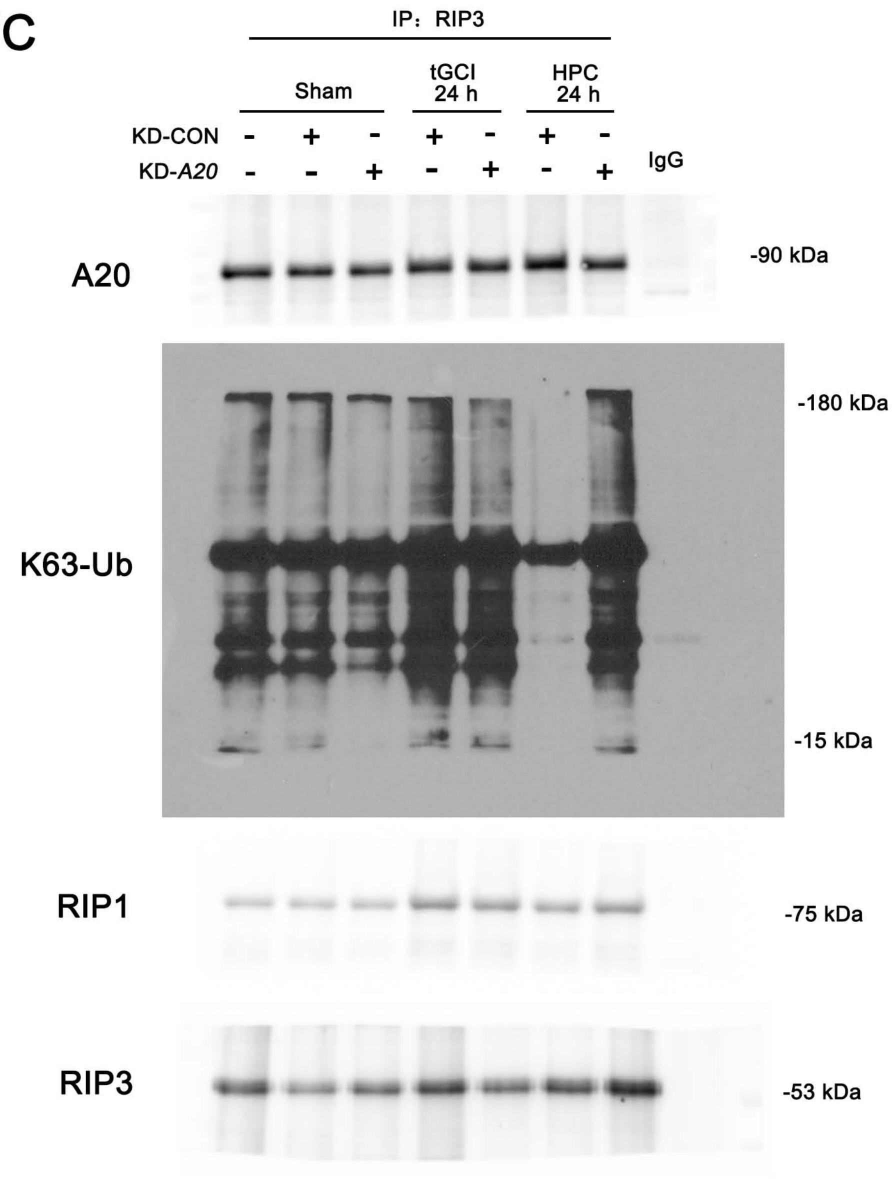
IP：RIP3
Sham
tGCI 24 h
HPC 24 h
KD-CON - + - + - + -
KD-A20 - - + - + - +
IgG
A20
-90 kDa
K63-Ub
-180 kDa
-15 kDa
RIP1
-75 kDa
RIP3
-53 kDa

Fig.7D

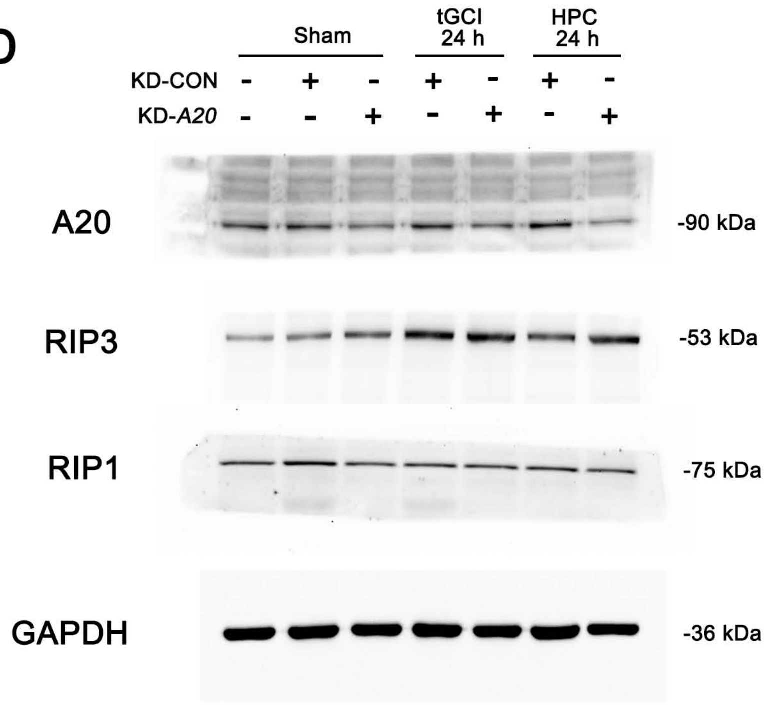
Sham
tGCI 24 h
HPC 24 h
KD-CON - + - + - + -
KD-A20 - - + - + - +
A20
-90 kDa
RIP3
-53 kDa
RIP1
-75 kDa
GAPDH
-36 kDa

Fig.8A

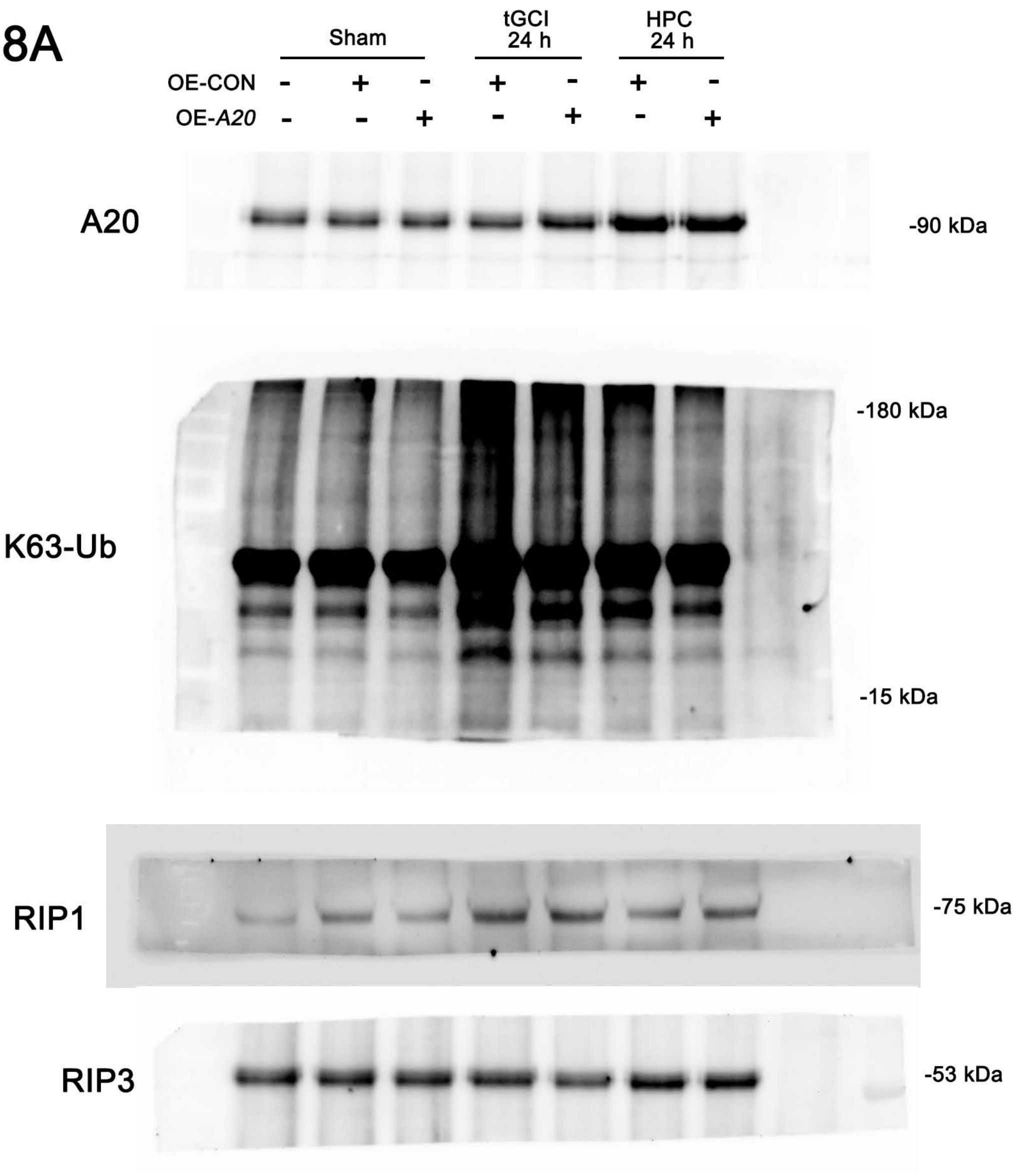
Sham
tGCI 24 h
HPC 24 h
OE-CON - + - + - + -
OE-A20 - - + - + - +
A20
-90 kDa
K63-Ub
-180 kDa
-15 kDa
RIP1
-75 kDa
RIP3
-53 kDa

Fig.8B

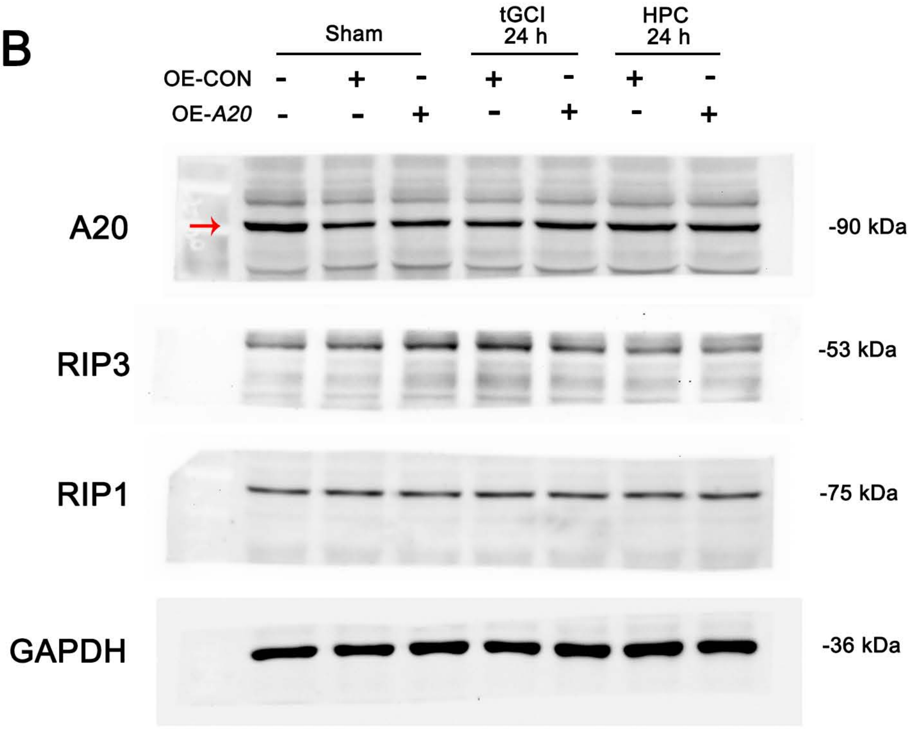
Sham
tGCI 24 h
HPC 24 h
OE-CON - + - + - + -
OE-A20 - - + - + - +
A20
-90 kDa
RIP3
-53 kDa
RIP1
-75 kDa
GAPDH
-36 kDa

Fig S1

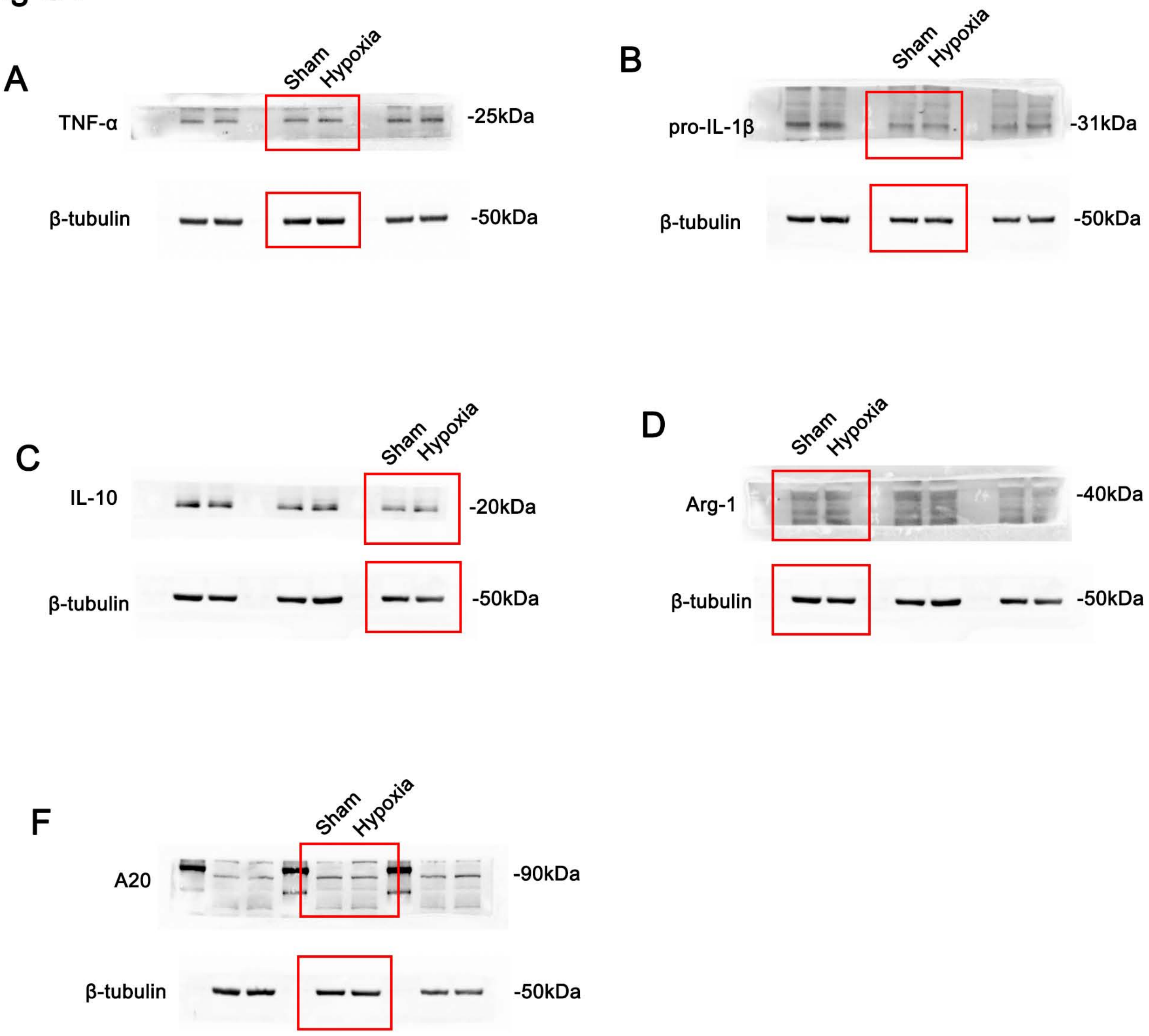
A
Sham
Hypoxia
TNF-α
-25kDa
β-tubulin
-50kDa
B
Sham
Hypoxia
pro-IL-1β
-31kDa
β-tubulin
-50kDa
C
Sham
Hypoxia
IL-10
-20kDa
β-tubulin
-50kDa
D
Sham
Hypoxia
Arg-1
-40kDa
β-tubulin
-50kDa
F
Sham
Hypoxia
A20
-90kDa
β-tubulin
-50kDa
